# Aminated Lignin‐Reinforced Biopolymer Hydrogels for Sustained Phosphate Delivery via Struvite Encapsulation in Acidic Environments

**DOI:** 10.1002/gch2.202500288

**Published:** 2025-09-24

**Authors:** Abrar Ali Khan, Arvind Singh Chandel, Vivek V. Ranade, Maurice N. Collins

**Affiliations:** ^1^ Stokes Laboratories School of Engineering Bernal Institute University of Limerick Limerick V94 T9PX Ireland; ^2^ Multiphase Reactors and Intensification Group Bernal Institute University of Limerick Limerick V94 T9PX Ireland; ^3^ Health Research Institute University of Limerick Limerick V94 T9PX Ireland; ^4^ SFI Centre for Advanced Materials and BioEngineering Research Dublin D02 PN40 Ireland

**Keywords:** aminated lignin, biopolymer hydrogel, controlled phosphate release, struvite encapsulation, sustainable agriculture

## Abstract

The escalating global demand for food production, coupled with excessive fertilizer use and freshwater depletion, necessitates sustainable solutions in agriculture. The excessive utilization of fertilizers to enhance crop productivity reflects a variety of negative impacts, including environmental and economic challenges. In this study, a biodegradable, dual crosslinked hydrogel composed of polyvinyl alcohol (P), chitosan (Chi), and aminated lignin (AL) is developed to encapsulate struvite (MgNH_4_PO_4_·6H_2_O), a slow‐release phosphate fertilizer. AL is synthesized via Mannich reaction using polyethyleneimine to enhance nitrogen content and functionality. The structural and functional characterization of the hydrogels is carried out using FTIR, SEM, XRD, and TGA. All pristine formulations exhibit high water‐holding capacity with non‐Fickian swelling behavior, reaching swelling values up to 706 ± 20.7%. Upon struvite loading, the swelling capacities reduce significantly, reflecting enhanced matrix density and encapsulation efficiency. Phosphate release studies in acidic citric solution (pH 3.3) show sustained release over 6−7 days. Kinetic modeling confirms a super case II transport mechanism (*n* > 1) and dominant diffusion‐controlled release (Higuchi model), while a poor fit to pseudo‐first‐order kinetics indicates nonconcentration‐dependent behavior. This study highlights the potential of lignin‐based hydrogels as eco‐friendly platforms for nutrient‐efficient fertilizer delivery, offering a promising pathway toward sustainable agriculture.

## Graphical abstract

The provision of adequate fertilizer in agricultural production is a global challenge. Here, a AL/Chi /P hydrogel is designed to release struvite fertilizer. These hydrogels show sustained release over 7 days. Modeling confirms case II transport and diffusion‐controlled release, highlighting the potential of lignin‐based hydrogels as eco‐friendly platforms for nutrient‐efficient fertilizer delivery, offering a promising pathway toward sustainable agriculture.

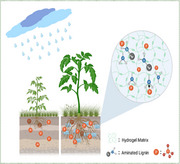

## 1 Introduction

World population is expected to reach 9.8 billion by 2050, which will ultimately demand increased crop production.^[^ ^1^ ^]^ The provision of adequate amounts of water and fertilizer in agricultural production is a challenge for achieving optimal plant growth and crop productivity.^[^ ^2^ ^]^ Soil salinization, desertification, urbanization, and industrialization are causing arable lands to shrink, and cultivating food from low‐fertile soils requires a large quantity of fertilizers to ensure food security. Due to overuse, a large proportion of fertilizer is lost to the environment (phosphorus: 80–90%, nitrogen: 40–70%), posing economic and environmental disquiets.^[^ ^3^ ^]^ Furthermore, the excessive freshwater usage in agriculture impairs the quality of freshwater in lakes, rivers, and aquifers among others.^[^ ^4^, ^5^, ^6^ ^]^ In short, proper fertilizer and water supply handling in agriculture are the two main factors influencing crop productivity and are thus essential for sustainable agricultural development and economic feasibility.

Phosphorus, a critical nutrient for plant growth, is mainly sourced from phosphate rock reserves. Due to fixation of phosphate in soil and surface runoff loss, large quantities of phosphate rock are mined and converted into phosphorus fertilizers to ensure high crop yields.^[^ ^7^, ^8^ ^]^ To overcome the dependency on finite and nonrenewable rock reserves for phosphorus, an alternative struvite (magnesium ammonium phosphate, MgNH_4_PO_4_) from waste sludge possesses promising characteristics, with low water solubility at neutral pH, economic feasibility and is identified as a source of phosphorus which can potentially minimize the negative environmental impacts.^[^ ^9^ ^]^ Furthermore, the hydrogel system, also called a “mini‐reservoir,” represents a burgeoning trend in agriculture with excellent water‐holding capacity and controlled‐release fertilizer efficiency, facilitating soil porosity and supplying oxygen to plant roots.^[^ ^10^ ^]^ However, most hydrogels commercially available in the market are based on synthetic polymers, for example, polyacrylamide, polyacrylic acid, sodium, or potassium polyacrylate, or derived from petroleum products, which are nonbiodegradable and pose sustainability concerns.^[^ ^11^, ^12^ ^]^ Henceforth, addressing the existing challenges and propelling the advancement of eco‐friendly agricultural products, the key lies in developing natural polymer‐derived hydrogels with controlled release properties, economic feasibility, while fostering harmonious coexistence between enhancing crop yield and environmental preservation.^[^ ^12^ ^]^ The utilization of hydrogel as a controlled‐release platform in agriculture has been well reported.^[^ ^13^, ^14^, ^15^, ^16^, ^17^ ^]^ Mentioning a few, Mengqiao Wu et al. reported Chi hydrogel membranes with iron, calcium and magnesium modified biochars embedded with N‐P‐K fertilizers. The hydrogels showed efficient slow‐release properties and promoted chilli plant growth.^[^ ^18^ ^]^ Other examples include starch‐grafted polyacrylamide hydrogels loaded with humic acid,^[^ ^19^ ^]^ rice straw graft copolymerized with acrylamide and N,N‐methylenebisacrylamide^[^ ^20^ ^]^ and lignosulfonate polymerized with methylacrylloxyethyl trimethyl ammonium chloride and acrylic acid,^[^ ^21^ ^]^ etc. Therefore, this study aims to develop a composite hydrogel, which has good water retention and slow‐release properties while preserving environmental health.

Biodegradable hydrogels are derived from natural sources such as lignin, cellulose, chitosan, starch, and alginate, which are cost‐effective and environmentally friendly.^[^ ^22^, ^23^ ^]^ Nowadays, extensive literature has been reported on fabricating lignin‐based hydrogels for controlled release and water retention applications in agriculture^[^ ^24^, ^25^, ^26^, ^27^, ^28^, ^29^ ^]^ owing to its abundant availability, renewable nature, and low‐cost extraction as a waste from biorefining industries such as ethanol, paper, and pulp industry.^[^ ^24^, ^25^, ^26^ ^]^ Additionally, the numerous active functional groups (aliphatic‐phenolic hydroxyl, methoxycarbonyl, carboxyl, etc) in lignin can be chemically modified for desired applications in the fields of fertilizers, soil amendments, heavy metal chelating agents, etc., among others.^[^ ^27^, ^28^, ^29^, ^30^ ^]^ The introduction of nitrogen‐containing functional groups in lignin using dimethylamine, ethylenediamine, diethylenetriamine, through amination (Mannich reaction) for the preparation of slow‐release nitrogen fertilizer (SRNFs) has been well documented in literature.^[^ ^30^, ^31^, ^32^, ^33^, ^34^, ^35^ ^]^ To increase the nitrogen content in lignin via Mannich reaction using polyethyleneimine (PEI) due to its many amino groups in its molecular chain is a promising way. These amino groups are protonated under lower pH to adsorb the target with a negative charge electrostatically.^[^ ^36^ ^]^ Additionally, Chi, a readily available natural polymer, is gaining attention as a promising material for applications in agriculture owing to biodegradability and nontoxicity.^[^ ^37^ ^]^ Furthermore, Chi plays a dual role in agriculture by acting as a bio‐stimulant and triggering plant defense mechanisms against biotic and abiotic stress, while also exhibiting antifungal, antibacterial, and antiviral properties against plant pathogens.^[^ ^38^, ^39^ ^]^ Moreover, Chi itself serves as a rich source of nitrogen (5.8–7.6%) and phosphorus (3.4–6.1%) for plants.^[^ ^40^, ^41^ ^]^ However, to overcome the poor mechanical stability of Chi hydrogel, incorporating PVA to bring mechanical stability is a promising constituent for several reasons, including nontoxicity and susceptible biodegradability among other characteristics.^[^ ^42^, ^43^, ^44^ ^]^ Furthermore, it is recognised and FDA‐approved as food additive E1203.^[^ ^43^, ^45^, ^46^ ^]^

To address these alarming environmental concerns and considering the favourable characteristics of lignin, Chi, and P, this study aimed to develop a dual crosslinked hydrogel with controlled fertilizer release properties. The as‐prepared P/Chi/AL hydrogel was laden with struvite, and the investigation includes the chemical and physical characteristics of the hydrogel, including FTIR, SEM, XRD, TGA, swelling characteristics, water retention capability, and phosphate release efficiency. This work will pave the way for chemically modified lignin‐based hydrogel as a slow‐releasing platform for the agricultural sector.

## 2 Results and Discussion

**Figure 1:**
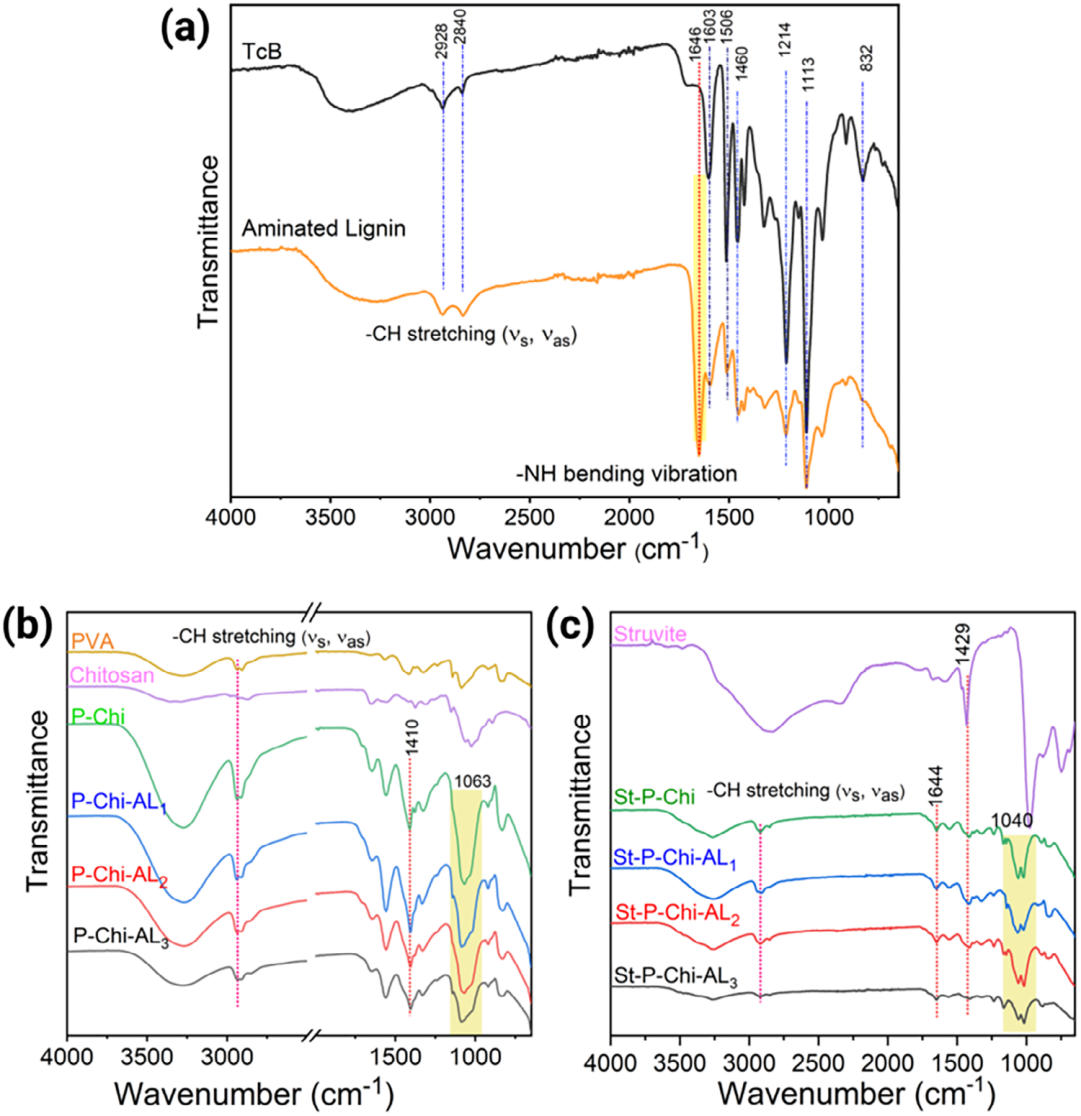
a) Comparison of FTIR spectra between kraft lignin and AL showing successful amination via the emergence of N─H bending vibrations at ≈1646 cm^−1^ and reduced aromatic skeletal peaks, b) Spectral changes in pristine hydrogel samples confirming imine bond (C═N) formation and acetal linkages, c) FTIR spectra of struvite‐loaded hydrogels indicate phosphate group interactions and hydrogen bonding, confirming successful struvite incorporation.

In this study, the chemical synthesis process of aminating lignin adopted the Mannich reaction under alkaline conditions as the core step to introduce amine groups (─NH_2_) into the molecular structure of lignin. The preparation process is shown in Figure S2 (Supporting Information). The infrared spectra of the pure lignin and aminated lignin clearly reflected the structure differences as shown in **Figure** **1**a. The broad peak at 3314 cm^−1^ corresponds to the aliphatic and phenolic hydroxyl groups, whereas the peak at 2928 and 2840 cm^−1^ are due to C─H asymmetrical and symmetric stretching vibrations in the methyl, methylene, and methoxy groups.^[^ ^47^ ^]^ Furthermore, the absorption peaks ≈1600 and 1515 cm^−1^ were attributed to the tensile vibration of the aromatic skeleton. In addition, the peaks ≈1460 and 832 cm^−1^ originates from C─H bending and out‐of‐plane deformation vibrations in lignin, respectively.^[^ ^48^ ^]^ It was suggested that the backbone structure of lignin was not disrupted by Mannich reaction as witnessed by peaks ≈1214 and 1113 cm^−1^ originating from guaiacyl, syringyl, and ether bonds in lignin structure, respectively.^[^ ^48^, ^49^ ^]^ However, some clear changes have been noted in the infrared spectra of AL, for example, the peak ≈832 cm^−1^ vanished in aminated lignin spectra. The intensity of peaks ≈1600, 1515, and 1460 cm^−1^, referring to C─H vibrations in the aromatic skeleton, decreased in the spectra of AL. The possible reason for this was Manich reaction occurred at the aromatic region of lignin structure. Furthermore, a new peak ≈1646 cm^−1^ was observed, originating from the N─H bending vibration in NH_2_ structure indicated that the amination reagents had been successfully grafted onto the lignin structure by Mannich reaction.^[^ ^50^ ^]^

**Figure 2:**
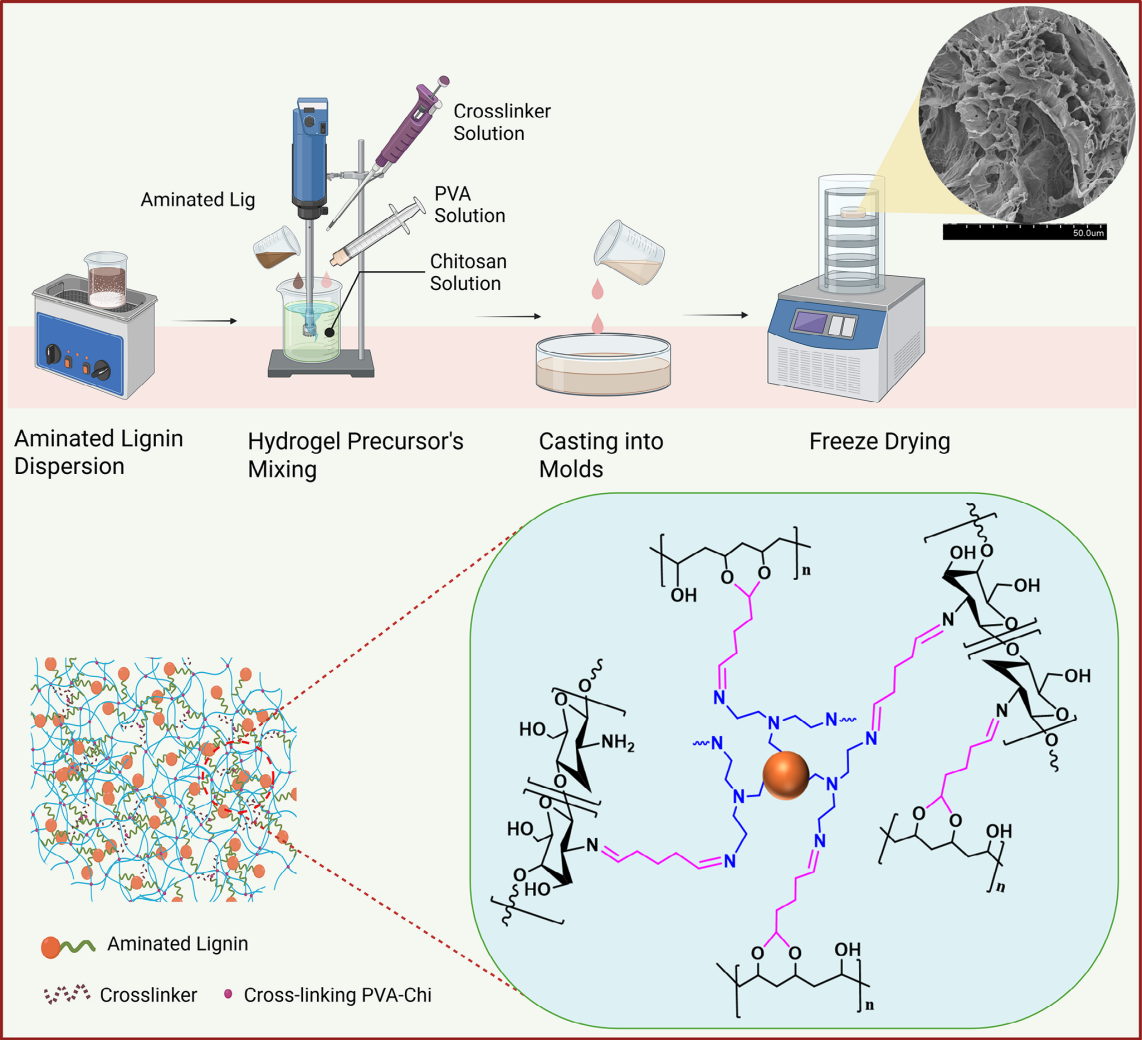
Schematic representation of P‐Chi‐AL_x_ hydrogel synthesis procedure and the corresponding reaction mechanism.

**Figure** **2** shows the schematic of the methodology employed in the synthesis of hydrogels and the corresponding reaction mechanism. The aminated lignin dispersion was prepared by sonication followed by the addition of Chi solution. After thoroughly mixing the AL/Chit solution was charged with PVA solution, and subsequently, GA solution was added as a crosslinking agent. The hydrogel network is formed due to the interaction of free amine groups of Chi and AL with the aldehyde group of GA, resulting in stable imine bond formation.^[^ ^51^ ^]^ These imine groups are formed because of a nucleophilic addition reaction, where the free amine groups of Chi and aminated lignin react with the aldehyde group of GA via Schiff base reaction, resulting in the formation of hemiaminal bridges (─C(OH)NHR)─) and removal of water.^[^ ^52^ ^]^ The free aldehyde groups on the other side of GA react with the hydroxyl groups of PVA via acetal bonding in an acidic medium (acetic acid), resulting in the formation of a stable hydrogel network even with a lower concentration of GA (0.24% v/v).^[^ ^53^ ^]^

Figure 1b,c presents the IR spectrum of pristine and struvite‐loaded hydrogels. The peaks ≈3270 and 1087 cm^−1^ are ascribed to the O─H and C─O stretching vibration, respectively, in the PVA chain.^[^ ^54^ ^]^ The characteristic absorption bands of Chi functional groups were observed at 1655 cm^−1^ (amide), 1422 cm^−1^ (CH_2_ bending), and 1023 cm^−1^ (C─O stretching).^[^ ^55^ ^]^ The bands ≈1410 cm^−1^ in pure P and in crosslinked hydrogels are due to characteristic C─H bending vibrations. With respect to pure P and Chi, significant changes in IR spectrum of crosslinked hydrogels are observed. For example, the peak ≈1560 cm^−1^ ascribed to the formation of imine bond (C═N) and symmetric deformation of NH^+3^ groups owning to the ionization of primary amine groups in the acidic medium.^[^ ^56^ ^]^ Furthermore, the peak at 1648 cm^−1^ can be assigned to C═N stretching vibrations. In addition, the acetal linkages were witnessed by the strong C─O─C stretching vibration at 1063 cm^−1^ owning to the reaction of hydroxyl groups of PVA with the GA.^[^ ^57^ ^]^ The struvite‐laden hydrogel showed visible variations in the IR spectrum, as shown in Figure 1c. The IR bands of pure struvite (data reused from our previous report);^[^ ^58^ ^]^ observed at 3415 to 2098 cm^−1^ are ascribed to H─O─H and ammonium ─NH stretching vibrations in struvite. The peak at 1431 cm^−1^ is due to NH_4_^+^ deformation, and band at 750 cm^−1^ is due to P─O─P stretching vibration. The strong vibration peak ≈976 cm^−1^ along with a shoulder ≈871 cm^−1^ are ascribed to the symmetric‐asymmetric stretching vibrations of the phosphate units (─PO_4_^−3^), and wagging modes of coordinated water, respectively.^[^ ^59^ ^]^ The IR spectrum of struvite loaded hydrogel showed noticeable shifts as shown in Figure 1c. Owning to the strong hydrophilic nature of P chain, numerous H‐bonding within P chain, AL, as well as among P, AL, Chi and struvite cause the broad *ν*O─H band stretching in pure struvite to be shifted to 3250 cm^−1^ in St‐P‐Chi‐AL_x_. Furthermore, the suppression of some IR bands (1600–1300 cm^−1^) and the appearance of low intensity band at 1234 cm^−1^ may be caused by the possible interaction of ─NH (struvite) with C─H of hydrogel matrix due to possible hydrogen bonding. In addition, the C─O─C stretching vibration peak are also observed to be shifted to lower wavenumber (1040 cm^−1^) accompanied by splitting into two lower intensity distinct bands, thus confirming the successful interaction of struvite in the hydrogel matrix.

**Figure 3:**
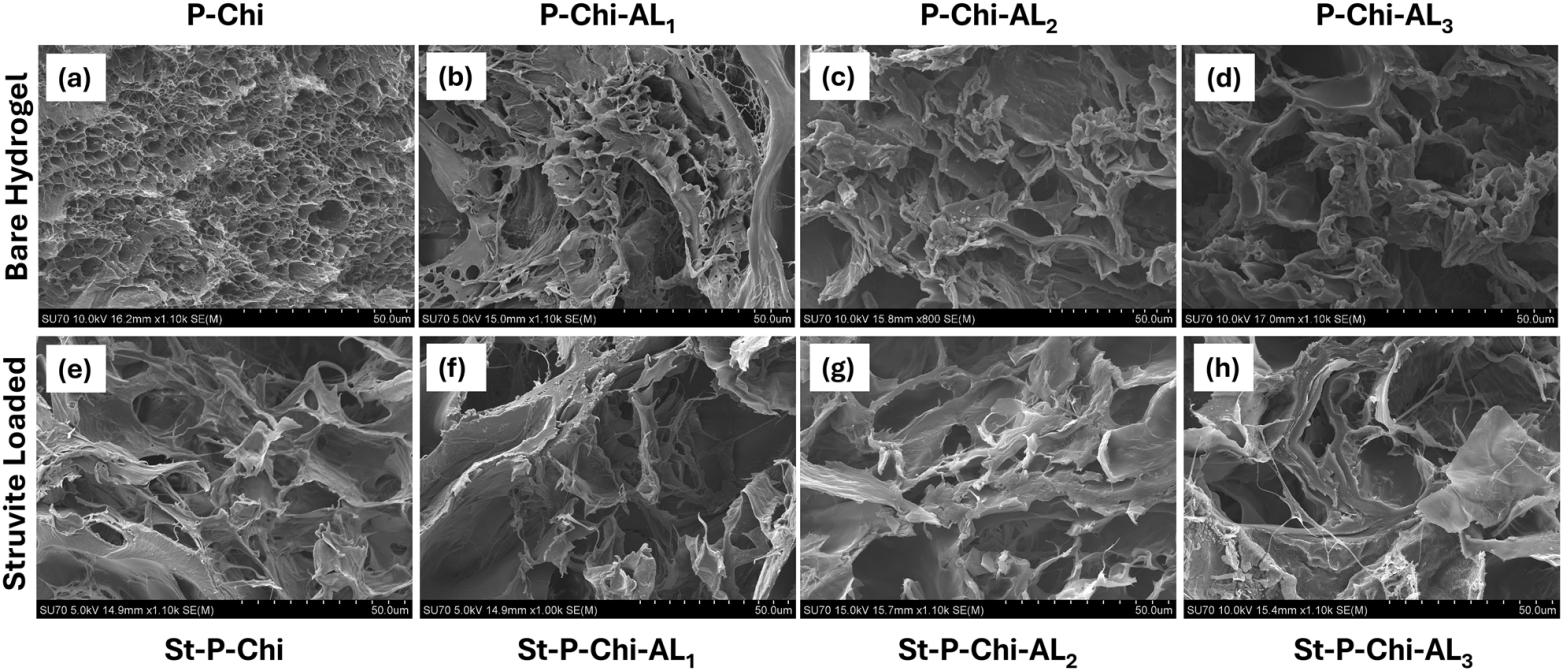
a–d) Pristine hydrogels show highly porous and interconnected structures, with increasing AL concentration leading to denser and smoother textures. e–h) Struvite‐loaded hydrogels demonstrate reduced porosity and embedded particles, confirming struvite dispersion. Higher AL content correlates with compact morphology and stronger matrix integration.

SEM analyzed the morphological analysis of pristine hydrogel and struvite‐loaded hydrogel, and the results are shown in **Figure** **3**. In general, all the hydrogels showed porous network structure; however, the porosity has significantly altered upon changing AL concentrations and struvite loading in the case of ladened hydrogels. For instance, the SEM image of P‐Chi hydrogel without AL and struvite showed a porous interconnected network structure with numerous voids (Figure 3a).^[^ ^60^ ^]^ After the addition of AL, all the hydrogels have retained their original porous structure, but rough and fragmented surfaces can be seen (Figure 3b–d), which suggests the integration of AL in the hydrogel matrix. At higher AL concentrations in P‐Chi‐AL_2_ and P‐Chi‐AL_3_ (Figure 3c,d), sheet‐like formation can be seen, which becomes more cohesive, compact and exhibits a refined structure at increased AL concentrations (Figure 3d). The struvite‐loaded hydrogels exhibited porosity; however, additional morphological changes can be observed upon the incorporation of struvite (Figure 3e–h). The porosity seemed to be decreased, as the struvite particles filled the pores, which led to a denser and more compact structure owing as shown in Figure 3g,h. At higher magnifications, struvite particles can be seen effectively embedded in the hydrogel matrix (Figure S3, Supporting Information). The increase in the cohesiveness and denser region is due to the strong hydrogen bonding interaction of the hydrogel matrix and struvite, also witnessed by FTIR analysis (Figure 1c), highlighting the successful incorporation of struvite. The higher AL concentration appeared to better integrate with struvite, allowing for more controlled release of phosphate.

**Figure 4:**
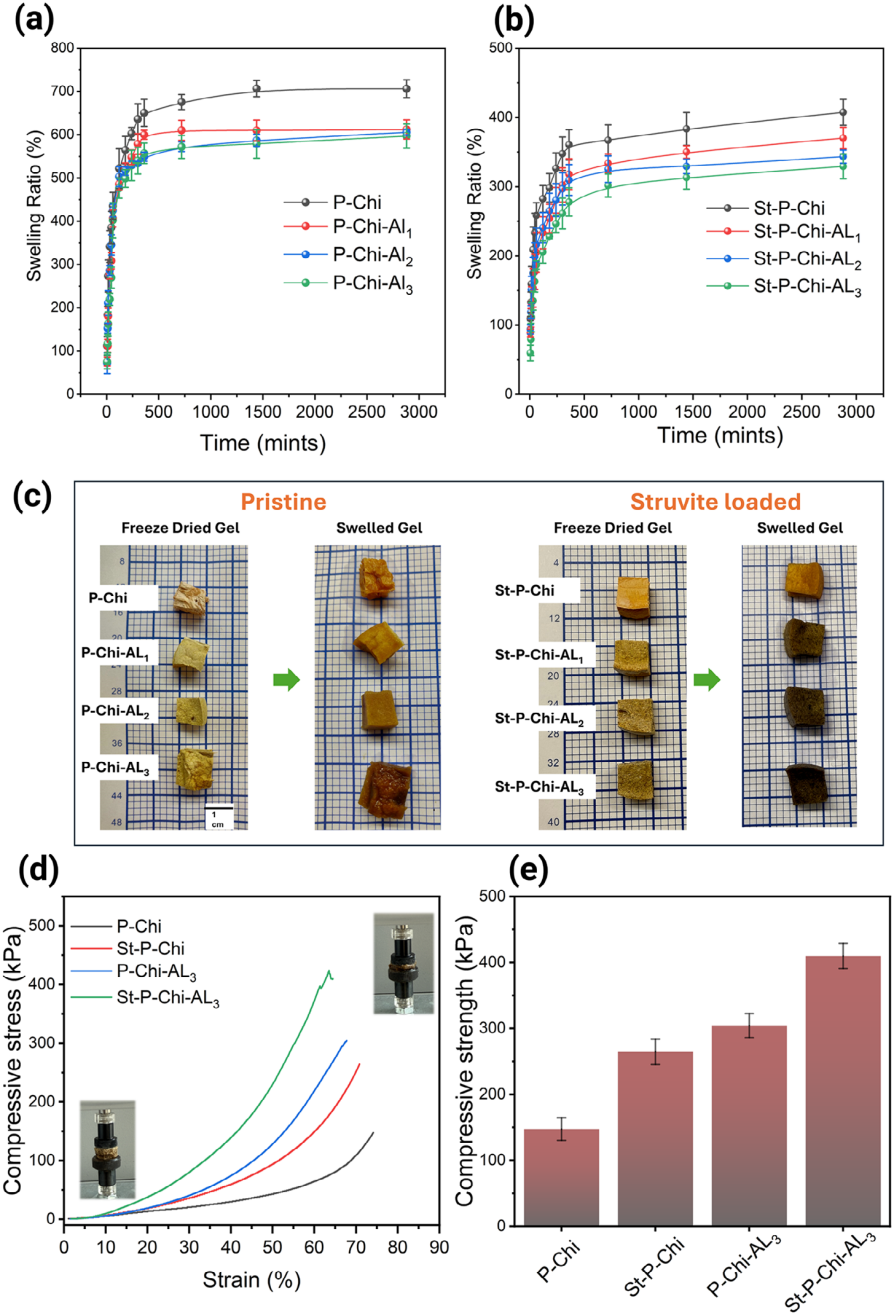
a,b) Time‐dependent swelling profiles of pristine and struvite‐loaded hydrogels. Increasing AL or struvite content decreases swelling due to stronger network cohesion. c) Digital images visually compare dry and swollen states, reinforcing the hydrogel's water‐absorbing potential. d,e) Compressive stress–strain curves and compressive strength (*n* = 3) of the hydrogels.

The water‐holding efficiency of hydrogel plays a pivotal role in agricultural applications. Hydrogels exhibit a nonuniform charge distribution due to the presence of numerous functional groups, and such charge asymmetry helps to form hydrogen bonds with water molecules and retain a significant quantity of water.^[^ ^61^ ^]^ The effect of variation in the amount of AL and incorporation of struvite in the case of ladened hydrogels on the water holding capacity is shown in **Figure** **4**. It has been observed that all synthesized hydrogels exhibited a swollen structure while accommodating large amounts of water at equilibrium swelling. It is worth mentioning that variation in the amount of AL and struvite loading significantly influences the water holding capacity of the hydrogels, ranging from 596 ± 27.7, 605 ± 14.1, 612 ± 22.1, and 706 ± 20.7, for P‐Chi‐AL_3_, P‐Chi‐AL_2_, P‐Chi‐AL_1_, and P‐Chi, respectively. However, upon loading struvite in the hydrogel matrix, the swelling capacities (%) gradually decreased to 329 ± 17.7, 343 ± 9.4, 370 ± 15.4, and 407 ± 18.7 for St‐P‐Chi‐AL_3_, St‐P‐Chi‐AL_2_, St‐P‐Chi‐AL_1_, and St‐P‐Chi, respectively. These hydrogels swell quickly upon immersing in water, and all the curves appear to reach a plateau, suggesting that the swelling process stabilizes after 6 h (Figure 4a,b). The phenomenon of a gradual decrease in water holding capacity upon increasing AL concentration could be explained by the fact that the amine groups of AL and Chi pose affinity toward the hydroxyl groups of P, leading to strong noncovalent interaction in the hydrogel matrix and thus give rise to a more compact structure (Figure 3) with less water holding capacity.^[^ ^62^, ^63^ ^]^

As the diffusion process involves the movement of suitable solvent into pre‐existing or dynamically formed spaces between macromolecular chains in the hydrogel matrix. Understanding the swelling process is crucial for developing hydrogels with controlled release applications. Using Korsmeyer–Peppas model, the diffusion exponent “n” and gel characteristic constant “k” have been calculated from the slope and intercept, respectively, of the plot of “ln Mt/M∞” versus ln “t” as shown in Figure S5 (Supporting Information) to analyze the mechanism of water uptake in these hydrogel matrices. The values of “n” and “R^2^” are tabulated in **Table** **1**. It was observed from the plot that the value of “n” corresponding to non‐Fickian‐diffusion mechanism (0.5 < n < 1). This type of diffusion mechanism involves the diffusion of water and polymer relaxation simultaneously. The experimental data aligned well with the nonlinear form of the Korsmeyer–Peppas model (Figure S5, Supporting Information). The presence of ─NH_2_ and ─OH functional groups in the hydrogel structure contributed to hydrogen bonding with water molecules, driving swelling behavior.

**Table 1:** Calculated equilibrium swelling percentages and diffusion exponent values using Korsmeyer–Peppas model for pristine and struvite‐loaded hydrogels.

Sample	Equilibrium swelling [%]	Diffusion exponent “n”	R^2^	Sample	Equilibrium swelling [%]	Diffusion exponent “n”	R^2^
P‐Chi	706 ± 20.7	0.89	0.97	St‐P‐Chi	407 ± 18.7	0.61	0.98
P‐Chi‐AL_1_	612 ± 22.1	0.97	0.95	St‐P‐Chi‐AL_1_	370 ± 15.4	0.63	0.97
P‐Chi‐AL_2_	605 ± 14.1	0.92	0.95	St‐P‐Chi‐AL_2_	343 ± 9.4	0.56	0.98
P‐Chi‐AL_3_	596 ± 27.7	0.97	0.96	St‐P‐Chi‐AL_3_	329 ± 17.7	0.67	0.98

For compressive strength analysis, one representative sample for AL added hydrogel (P‐Chi‐AL_3_) and one representative sample struvite loaded hydrogel (St‐P‐Chi‐AL_3_) were compared with their respective mother samples (P‐Chi and St‐P‐Chi) and the results obtained are presented in Figure 4d,e. As strength of the hydrogel is crucial for evaluating the hydrogels’ resilience during handling. It was observed that the compressive strength of hydrogel after AL addition significantly increased from 147.52 ± 17.2 to 304.34 ± 18.1 kPa for P‐Chi and P‐Chi‐AL_3_, respectively. This might be due to an increase in hydrogel matrix's density and compactness afterAL. Similarly, the compressive strength was further increased after struvite loading in the hydrogel matrix. For example, after the addition of struvite, the compressive strength observed for St‐P‐Chi and St‐P‐Chi‐AL_3_ were 264.53 ±19.2 and 409.93 ±19, respectively. These results demonstrate the significant role of both AL and struvite in improving the mechanical properties of the hydrogels, which directly impacts their handling and performance in agricultural applications. However, it's important to note that the increase in compressive strength (matrix density) did not significantly reduce the swelling capacity of the hydrogels, as illustrated in Figure 4a,b. This suggests that the hydrogels maintain their ability to absorb water despite the increased matrix density, ensuring their suitability for agricultural use where water retention is essential.

**Figure 5:**
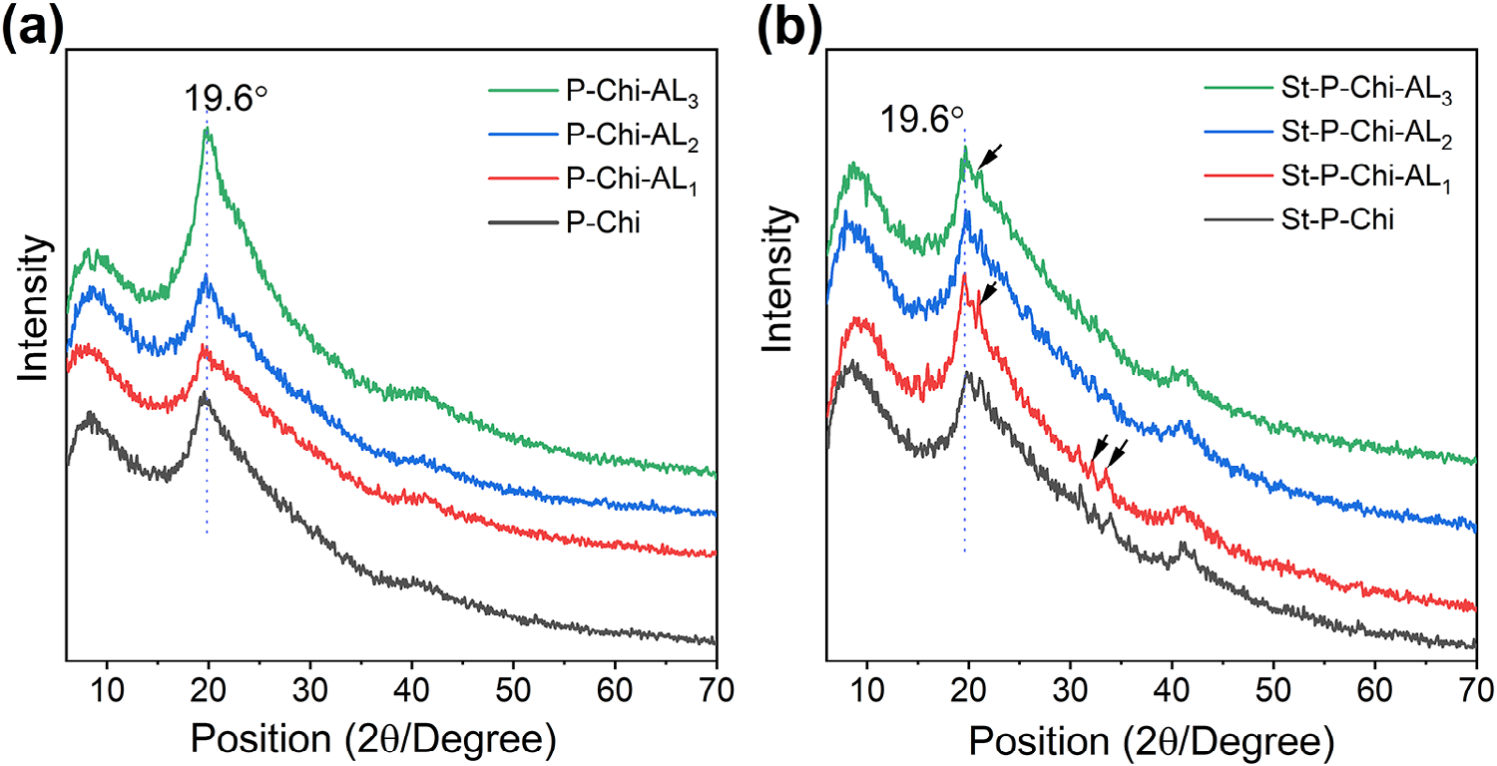
XRD analysis of hydrogels. a) Disappearance of individual crystalline peaks of P and Chi in pristine hydrogels suggests strong miscibility and network formation. b) Presence of characteristic struvite diffraction peaks in loaded hydrogels confirms encapsulation without disrupting the hydrogel's amorphous matrix.

Furthermore, the wide‐angle XRD pattern of hydrogel precursors and as‐prepared hydrogels is depicted in Figure S3 (Supporting Information) and **Figure** **5**, respectively. In the range of 10°–50°, XRD pattern of PVA shows a strong diffraction peak owning to the semicrystalline nature at 2θ value of 19.5° indexed as 101 lattice planes. This peak is often employed for the investigation of variation in the crystallinity of P. The semicrystalline peak is followed by a shoulder at 2θ value of 22.8° indexed as 201 lattice planes. Diffraction maxima observed at 2θ value of 40.8° are ascribed to lattice plane 100.^[^ ^64^, ^65^ ^]^ The XRD pattern of pure Chi as depicted in Figure S3 (Supporting Information) reveals the crystalline characteristic of Chi, and the peak could be observed at 2θ value of 20° and a small peak at 9.4°.^[^ ^66^ ^]^ However, the diffractogram of AL revealed the lignin remain amorphous even after Manich reaction and no significant variation were observed in the broad amorphous peak of lignin around 2θ of 20°. The diffractogram of pristine hydrogel and struvite ladened hydrogel are presented in Figure 5a,b. For a mixture of each component, i.e., P, Chit and AL, each component should have its own crystal peak in the blend hydrogels. However, the Chi XRD peak at 2θ value 20°, P XRD peak at 2θ value of 22.8° and 40.8° disappeared. In addition, the Chi diffractogram peak at 9.4° became broad while those of P at 2θ value of 19.5° became weak in the hydrogel blend. These results suggest that P, Chit and AL are compatible, which leads to forming a porous hydrogel network.^[^ ^67^ ^]^ Notably, the diffractogram of struvite‐laden hydrogel, as shown in Figure 5b, indicates that the incorporation of struvite in the hydrogel matrix acts as a filler and does not significantly alter the crystallinity. However, some low intensity new diffraction peaks owning to struvite are observed at 2θ value of 15°, 21°, 27.35°, 30.91°, 32.08°, and 33.42°.^[^ ^68^ ^]^ These results suggest the good compatibility P, Chi, AL and struvite in the hydrogel platform.

**Figure 6:**
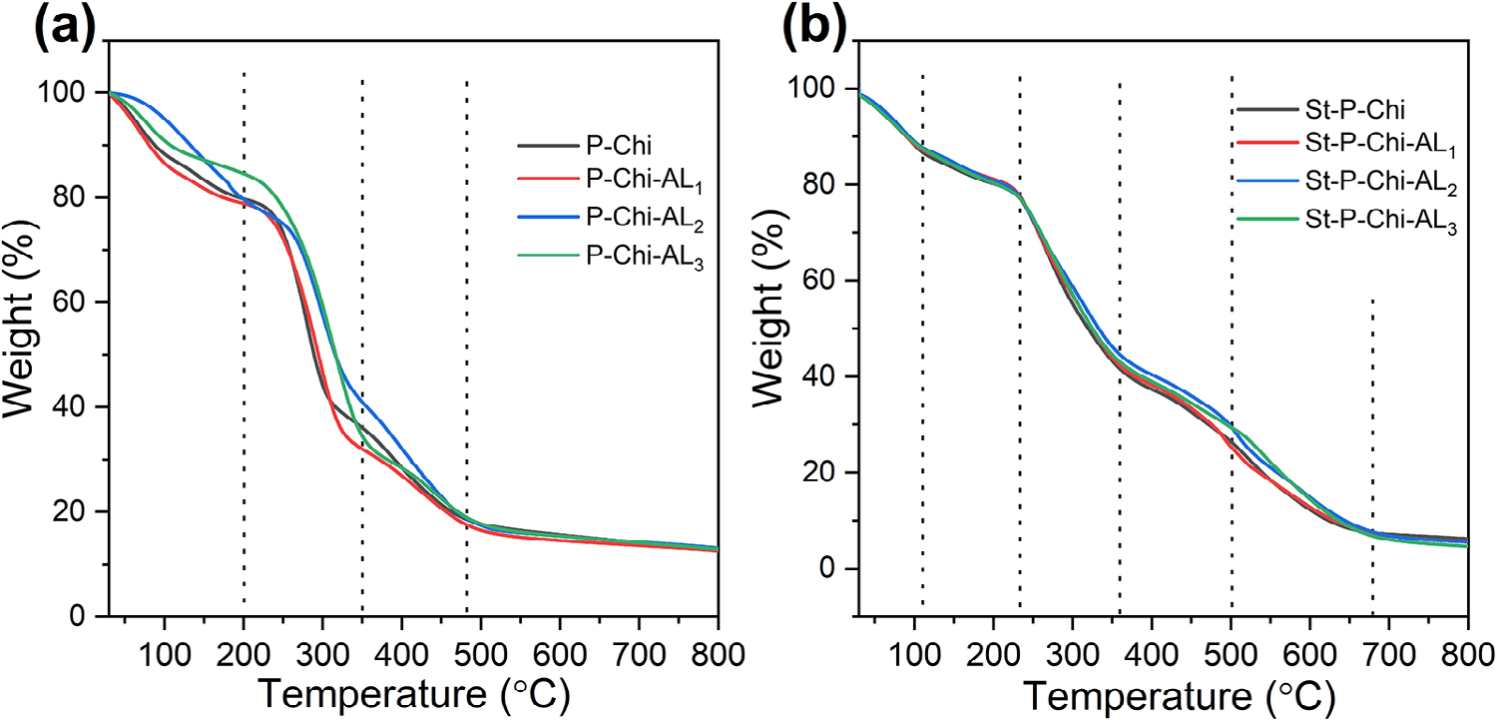
a) TGA profiles of pristine hydrogels showing multistep thermal degradation related to component breakdown. b) Struvite‐loaded samples display earlier degradation onset due to struvite decomposition, with improved thermal compatibility evident from shifted Tmax.

Furthermore, to investigate the thermal stability of the hydrogels, thermogravimetric analysis was performed, and the effect of variation in the AL concentration and struvite addition on thermal degradation and stability of the blend hydrogel is presented in **Figure** **6**. The peak values of DTG and residual percentage at 800 °C are summarized in **Table** **2**. The thermal degradation process of pristine hydrogel showed four steps with no significant weight loss till 200 °C. The first small amount of weight loss (70–110 °C) was due to the amount of absorbed moisture owing to the strong hydrophilic nature of PVA. During the second step (200–350 °C) the initial thermal decomposition of hydrogel started which is the primary degradation stage of pristine hydrogel. This maximum degradation step could be attributed to the complex degradation process of P, Chi and AL framework including the dehydration of saccharides rings in Chi and depolymerization of AL units and breaking of connection bonds.^[^ ^69^, ^70^ ^]^ In the third degradation step (370–500 °C) the thermal decomposition of some of the by‐products generated by P chain occurs, while the fourth step beyond 500 °C is the carbonization stage. After adding struvite in the hydrogel matrix, the thermal stability was significantly altered, as shown in Figure 6b. The thermal degradation is divided into five steps. In the first step below 100 °C the thermal evaporation occurs followed by the decomposition of struvite embedded in the hydrogel matrix (160 °C). The increase in the T_max_ of struvite from 100 °C as reported by Ramlogan et. al.,^[^ ^71^ ^]^ to 160 °C could be attributed to the excellent compatibility of struvite in the hydrogel blend, which is due to strong hydrogen bonding. Figure S4 (Supporting Information) summarizes changes in the peak temperature from DTG analysis after struvite loading in hydrogels.

**Table 2:** DTG peak temperature and residue (%) after TGA at 800 °C.

Sample	Peak temperatures [°C]				Residue [%] at 800 °C
	Peak 1^st^	Peak 2^nd^	Peak 3^rd^	Peak 4^th^	
P‐Chi	70	280	388	–	12.57
P‐Chi‐AL_1_	70	296	422	–	12.63
P‐Chi‐AL_2_	78	297	420	–	13.09
P‐Chi‐AL_3_	80	318	440	–	12.91
St‐P‐Chi	85	160	268	505	6.22
St‐P‐Chi‐AL_1_	85	160	254	490	5.52
St‐P‐Chi‐AL_2_	85	160	256	508	5.56
St‐PVA‐Chi‐AL_3_	85	160	281	544	4.61

**Figure 7:**
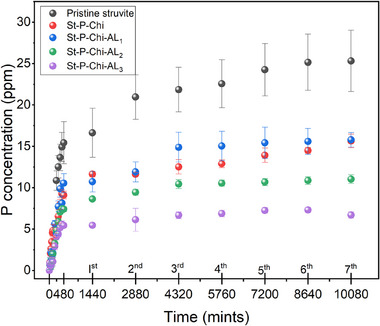
Graph depicting the cumulative phosphate release in acidic conditions. The release follows a biphasic pattern initial burst followed by controlled diffusion‐demonstrating the hydrogel's ability to moderate nutrient delivery. Error bars indicate consistency across replicates.

In phosphorus‐stressed conditions, plants release organic acids, for example, oxalic acid, malic acid, and citric acid, and the latter have been shown to induce higher phosphorus mobilization.^[^ ^72^ ^]^ In this report, citric acid solution (50 ppm, pH 3.46) was selected as a typical organic acid to study the controlled release behavior of phosphate from struvite encapsulated in a hydrogel matrix (**Figure** **7**). Each data point represented in the figure is the average value of three measurements with a standard deviation. The phosphate from the hydrogel matrix is closely related to the swelling characteristics, which are also influenced by the functional groups present. A fast solubilization and release behavior could be observed for in the first 8 h of the experiment. This abrupt release on the first day may be due to the release of phosphate from struvite embedded in the surface of the hydrogel matrix. Since struvite is readily soluble at lower pH, the hydrogel matrix's contribution to the fertilizer solubilization process is insignificant, acting instead as a barrier to the fast diffusion and thus enhancing the sustained release. In addition to providing the physical barrier, there is an electrostatic interaction between the amines and phosphates because the polyethyleneimine is a cationic polymer with many amino groups in its macromolecular chains that can be easily protonated in an acidic medium to adsorb phosphate with a negative charge electrostatically.^[^ ^73^ ^]^ In acidic conditions, the polyethyleneimine chain elongates and acts as a proton sponge. The elongation of the polyethyleneimine chain in an acidic medium is explained by the fact that, as the system progresses from unprotonated to protonated, a gradual increase in electrostatic interactions occurs. This causes repulsion among the monomeric units, causing the polyethyleneimine chain to extend.^[^ ^74^ ^]^ Due to this nature of elongation and protonation, significant controlled release could be observed. To provide a comparative analysis of our slow‐releasing platform's efficiency against other recently reported slow‐release systems, we have compiled data from recent studies in **Table** **3**.

**Table 3:** Comparison of phosphate release efficiency in slow‐release fertilizer hydrogel with hydrogel reported in this work (data from ScienceDirect using the keyword “Phosphorous slow‐release fertilizer” acquired in August 2025).

No #	Composition	Fertilizer type	Mechanism of loading	pH	Slow‐release efficiency	Refs.
1	Cellulose, Iron hydroxide NPs	Phosphate	Adsorption from wastewater	–	–	[75]
2	Lignin, Acrylic acid, METAC	NH_4_^+^, H_2_PO_4_^−^	Adsorption	7	30 days in soil	[76]
3	HTC, HA, PVA	Phosphorous	Impregnation	–	28 days	[77]
4	Chitosan, Metal modified biochar	NPK	Encapsulation	–	33 days in soil	[78]
5	Montmorillonite‑sodium alginate, chitosan	Phosphorous	Adsorption	9	19 days	[79]
6	guar gum‐g‐poly methyl methacrylate‐cl‐polylactic acid	AP and DAP	Adsorption	–	62.27% and 53.75%	[80]
7	Lignin, PVA	Struvite (phosphorus)	Encapsulation	3	>7 h	[81]
8	AL, PVA, chi	Struvite (phosphorus)	Encapsulation	3.46	>8 days	This study

Furthermore, the phosphate release kinetics were analyzed using Korsmeyer–Peppas, Higuchi, zero order and pseudo‐first order kinetic models and the coefficient of determination “R^2^” values are presented in **Table** **4** and Figure S6 (Supporting Information). For all samples, the nonlinear fits yielded higher R^2^ values than their linear counterparts, suggesting that the release process deviates from simple linear kinetics and may involve complex diffusion and polymer relaxation processes. Among the models studied, the nonlinear Higuchi kinetic models revealed excellent fitting, suggesting that the release of phosphate undergoes a diffusion‐controlled mechanism within the hydrogel matrix, which is in agreement with the Higuchi theory for diffusion‐controlled release from polymeric systems.^[^ ^82^, ^85^ ^]^ In addition, the zero‐order kinetic model further suggested a nearly constant phosphate release rate. Furthermore, the Korsmeyer–Peppas model revealed moderate R^2^ values for all the hydrogels, suggesting that the phosphate release involves the combined involvement of diffusion and matrix relaxation mechanisms (“n” > 1) as shown in Figure S6 (Supporting Information). During the initial phase of release, polymer relaxation tends to play a more significant role as the network begins to expand and create voids, facilitating the initial burst release of phosphate. As the hydrogel continues to swell and the system reaches equilibrium, the role of diffusion becomes more prominent, governing the sustained release phase. The balance between these mechanisms determines the overall release profile, and as seen in the experimental data, the hydrogel follows a super case II diffusion mechanism, indicating a dominant interaction between both processes (Figure S6a, Supporting Information).

**Table 4:** Kinetic study of phosphate release from hydrogel using implemented kinetic models.

Sample	Korsmeyer–Peppas kinetic model [R^2^]		Higuchi kinetic model [R^2^]		Zero‐order kinetic [R^2^]		Pseudo‐first order kinetic model [R^2^]	
	Linear	Logarithmic	Linear	Logarithmic	Linear	Logarithmic	Linear	Logarithmic
Pure struvite	0.73	0.84	0.75	0.92	0.61	0.92	0.34	0.73
St‐PVA‐Chi	0.86	0.94	0.83	0.97	0.71	0.97	0.45	0.86
St‐PVA‐Chi‐AL_1_	0.84	0.91	0.82	0.94	0.67	0.94	0.42	0.84
St‐PVA‐Chi‐AL_2_	0.74	0.86	0.76	0.94	0.59	0.94	0.30	0.73
St‐PVA‐Chi‐AL_3_	0.76	0.85	0.67	0.88	0.51	0.88	0.33	0.76

## 3 Conclusion

In this study, a dual crosslinked hydrogel system was successfully developed using P, Chi, and AL (AL), with glutaraldehyde (0.24% v/v) as the crosslinking agent. The influence of AL concentration on the hydrogel's structural, morphological, thermal, swelling, and nutrient release characteristics was systematically investigated. A clear inverse relationship was observed between AL content and swelling behavior, with equilibrium swelling percentages decreasing from 706 ± 20.7% to 596 ± 27.7% as AL content increased. Upon encapsulation of struvite, the swelling capacity further decreased (407 ± 18.7% to 329 ± 17.7%), indicating enhanced matrix compactness and efficient nutrient incorporation. All hydrogels showed excellent thermal stability. The release profile of the guest molecule (P) in citric acid solution exhibited sustained release over a period of 6 to 7 days. Different kinetic models were employed, and it was found that all the struvite‐loaded hydrogels followed super case II diffusion transport “n” > 1 for phosphate‐controlled release (Korsmeyer–Peppas model). Furthermore, it was also found that the sustained release of phosphate from all hydrogels was governed by combined polymer relaxation and diffusion mechanisms (Higuchi model). However, the release was not predominantly concentration dependent (lower R^2^ values of the pseudo‐first order kinetic model). Overall, the PVA–chi–AL hydrogel system demonstrates strong potential as a cost‐effective, biodegradable platform for slow‐release phosphate fertilizers. Beyond its laboratory‐scale performance, this work contributes to the broader goal of sustainable agriculture by utilizing renewable resources such as lignin and struvite, reducing dependence on nonrenewable phosphate rock, and promoting environmentally friendly nutrient management. The modular hydrogel design also offers future adaptability for multi‐nutrient delivery, making it a promising candidate for precision and climate‐resilient farming applications.

## 4 Experimental Section

### Materials

Materials Kraft lignin (TcB) with a molecular weight (Mw) of 3153 g mol^−1^ was supplied by Tecnaro (GMbH, Ilsfeld, Germany). Analytical grade Sodium hydroxide (NaOH) pellets with purity ≥ 98% were purchased from AppliChem GmbH (Ilsfeld, Germany). Poly(vinyl alcohol) (PVA, 99% hydrolyzed) with a Mw of 85 000−124 000 g mol^−1^, Formaldehyde, Magnesium Ammonium Phosphate (Struvite), and Chitosan (medium molecular weight, deacetylated chitin) were purchased from Sigma−Aldrich. Polyethyleneimine branched (Mn 60 000 g mol^−1^) 50 wt% aqueous solution was purchased from Thermo Scientific. Glutaraldehyde solution (≈50% in H_2_O) was purchased from Fluka Analytical. All chemicals were used without any further purification.

### Lignin Amination with PEI (AL)

Lignin amination was achieved through the Manich reaction. Briefly, 1 g of kraft lignin (TcB) was added to 25 mL NaOH solution (1 m, pH 13–14) and kept under stirring overnight at 60 °C. After complete dissolution of lignin, the solution was charged with 2.5 mL (1.28 g) of PEI (50% solution in water) dropwise and allowed to stir for the next 30 min. In the next step, the solution's temperature was raised to 80 °C with dropwise addition of 1 mL formaldehyde and allowed to stir for the next 4 h at ambient rpm. Finally, the AL was collected and washed several times with deionized water through centrifugation to remove the unreacted materials, followed by freeze drying for further use.

### Synthesis of P‐Chi‐AL Hydrogels

P‐chi AL hydrogel was fabricated by chemical crosslinking method using glutaraldehyde as a crosslinker, followed by freeze‐thaw process. Briefly, a stock solution of P (16 w/v %) was formed by taking the corresponding amount of P polymer in cold deionized water (solution A). The mixture was first stirred for 15 min to avoid lumps formation, and then the temperature was raised to 90 °C using a heat plate and a vortex stirrer for 4–5 h until a clear viscous solution was obtained. The as‐prepared P solution was incubated (30 °C) for later use. Another stock solution (solution B) of Chi (2 w/v %) was prepared by dissolving the corresponding amount of Chi in 1.5% (v/v %) of acetic acid solution. For hydrogel preparation, the corresponding amount of AL was first well dispersed in DOI water using an ultrasonic bath, followed by the addition of Chi solution under stirring. In the next step, the solution was charged with P solution (hot, to avoid hydrogen bonding) and allowed to stir for several minutes. Afterwards, the corresponding solution of crosslinker (GA) was added, and the resulting mixture was kept under stirring for some time before pouring it into moulds. These molds were first lifted overnight at room temperature and then frozen at −20 °C for 12 h, followed by thawing at room temperature for complete crosslinking to form aminated lignin AL‐derived hydrogels. Finally, the wet gel was washed with ethanol to remove the unreacted substances. In case of struvite‐loaded hydrogels, all the procedures were kept the same except the addition of struvite 0.1 g in the hydrogel matrix. The prepared hydrogels were denoted as P‐Chi‐AL_x_ for pristine hydrogels and St‐P‐Chi‐AL_x_ in case of struvite‐loaded hydrogels, where “x” indicates the weight of AL (x = 0.1, 0.2, 0.3 g) in the hydrogel matrix. All the formulations of pristine and struvite‐loaded hydrogels have been summarized in Table S1 (Supporting Information).

### Characterization Techniques

The surface functional group analysis of freeze‐dried hydrogels was performed by FTIR spectroscopy using the PerkinElmer Spectrum 100 Spectrometer accompanied by an attenuated total reflectance (ATR) accessory. A total of 10 scans were performed for each analysis in a range between 4000 and 650 cm^−1^. Furthermore, the scanning electron microscopy (SEM) images of the freeze‐dried samples, were acquired using an SEM‐SU70 Hitachi (Tokyo, Japan), under accelerating voltage of 5–10 kV, and work distance 15 ± 2 mm. X‐ray diffraction analysis (XRD) was performed employing PANalytical Empyrean instrument operating at 40 kV and 40 mA provided with a monochromatic Cu‐K α radiation source (*λ* = 1.5418 Å). The data was collected in a range of 5–55 at room temperature, provide with soler slit of 0.04 rad, divergence slit of 1/8°, mask 10, Anti‐scatter slit of 1/4ο with step size 0.026 and time per step 80 s were employed.

Simultaneous measurement of weight change (TGA) was performed using a thermal analyzer (TGA 4000 PerkinElmer). Approximately 10 mg of samples were placed in a Teflon crucible, and all the analysis was performed under an inert atmosphere with continuously flowing nitrogen gas at a rate of 40 mL⋅min^−1^. During the analysis, the temperature was raised from 30 to 900 °C at a heating rate of 20 °C min^−1^. The compressive strength of equilibrium swollen hydrogels was measured using an uniaxial IMADA Force–Displacement Measurement unit. A speed of 1 mm min^−1^ and measuring speed 0.5 mm min^−1^ was used for compression of the samples between two circular plates and at least three runs were carried out on each sample, with the mean ± standard deviation presented in the results. The equations used to determine the gel strength and stress–strain plot of the samples from the data obtained were as follows:

(1) $$\mathrm{Stress}\;\;\left(\mathrm{strength}\right)=F/A$$

(2) Strain%=ΔL/L∗100

where, F denotes the force (N), A is the cross‐sectional area of the hydrogel (m^2^), L is the original height (m), and ∆*L* is the change of height (m).

### Characterization Techniques—Equilibrium Swelling Studies and Diffusion Coefficient

The percentage swelling at regular intervals was measured in deionized water. Before weighing, the wet gels were blotted on filter paper to remove surface water. The hydrogel percentage swelling was calculated by Equation (1):

(3) $$swelling=\left[Mt-Mo/Mo\right]\;*100$$

In Equation (1) “Mt” is the weight of wet hydrogel and “Mo” is the weight of dried hydrogel. To analyze the theoretical water uptake analysis to calculate the Fickian, Non‐Fickian and/or Case II diffusion mechanism, the portion water absorption curve with a fractional water uptake (Mt / M∞) was studied using  the Korsmeyer–Peppas model (Equation 4).^[^ ^83^, ^84^ ^]^

(4) $$\frac{Mt}{M\infty \;}=K_{kp}t^{n}$$

where, “Mt” and “M∞” is the amount of water taken at time “t” and at equilibrium, respectively; however, “K_kp_” is gel characteristic constant while “n” shows the mechanism of transport of the penetrant. The value of constants “n” and “K” can be calculated from the slopes and intercepts, respectively, of the plots of ln(M_t_/M_∞_) versus ln“t” from the experimental data (logarithmic fit). For 0.5 < *n* < 1, the process is governed by a non‐Fickian diffusion mechanism, whereas *n* < 0.5 indicates a Fickian diffusion mechanism and “n” = 1.0 is for case II type diffusion and “n” > 1 is for super case II.^[^ ^83^ ^]^

### Characterization Techniques—Phosphate Release Study

The phosphate release study from the hydrogel platform was performed in static mode in triplicate by taking 50 mg of room temperature dried hydrogel using a 50 mL tube filled with 50 ppm citric acid solution having a pH of 3.46. For reference the phosphate release from pure struvite was studied as reference under the same conditions. The tubes were kept static in a tube rack in SciQuip Incubator (Incu‐160C) with controlled temperature of 25–30 °C. Two milliliter Aliquots were collected by slightly shaking the tube after the specific time intervals and replenished with 2 mL citric acid solution (50 ppm). For phosphate determination as “P” through ICP, 1 mL aliquot of sample was diluted to 5 mL with 1 m HNO_3_. The phosphate release kinetics were studied using Korsmeyer–Peppas model (Equation 5), Higuchi model, Zero‐order kinetic model, and pseudo‐first‐order kinetic model. The Higuchi model can be articulated as follows.

(5) $$\frac{M_{t}}{M_{\infty }\;}=K_{H}t^{1/2}$$

where “M_t_” represent the amount of phosphate release at time “t”, “M_∞_” represent the maximum amount of phosphate release, and “K_H_” is the Higuchi constant. The data were plotted against the square root of time as the cumulative percentage of phosphate release. The Higuchi model describes the nutrient release from insoluble matrix as the square root of a time‐dependent process and can be applied to water‐soluble and poorly water‐soluble nutrients. In zero‐order kinetic model as shown in Equation (6).

(6) $$\frac{M_{t}}{M_{\infty }}=K_{o}t$$

“K_ο_” is the zero‐order release constant and the graph can be analyzed plotting cumulative release against time. The pseudo‐first order kinetic models, as shown in Equation (7), can be applied to analyze the nutrient release from porous matrices.

(7) $$\frac{M_{t}}{M_{\infty }}=1-e^{-K_{1}t}$$

where “K_1_” is the pseudo‐first‐order rate constant, and the graph can be obtained plotting by log cumulative release percentage against time “t”.

## Conflict of Interest

The authors declare no conflict of interest.

## Author Contributions

A.A.K. performed methodology, investigation, formal analysis, data curation, conceptualization, and wrote the original draft. A.S.C. performed data curation and conceptualization. V.V.R. performed supervision, acquired resources, and administered the project. M.N.C. performed supervision, acquired resources, and administered the project.

## Supporting information



Supporting Information

## Data Availability

The data that support the findings of this study are available from the corresponding author upon reasonable request.

## References

[1] United Nations Department of Economic and Social Affairs, 2017. “World Population Projected to Reach 9.8 Billion by 2050 and 11.2 Billion by 2100.” June 21, 2017.

[2] N. Sarhan , E. G. Arafa , N. Elgedawy , K. N. M. Elsayed , F. Mohamed , Sci. Rep. 2024, 14, 1. 38951590 10.1038/s41598-024-58875-1 PMC11217492

[3] I. Rashmi , T. Roy , K. S. Kartika , R. Pal , V. Coumar , S. Kala , K. C. Shinoji , in Contaminants in Agriculture: Sources, Impacts and Management, Springer, Germany 2020, pp. 3–41.

[4] J. Mateo‐Sagasta , S. Zadeh , H. Turral , in More People, More Food, Worse Water?: A Global Review of Water Pollution from Agriculture, Food and Agriculture Organization of the United Nations and International Water Management Institute, United Nations 2018.

[5] Chai , Y. J. Pannell , D. G. Pardey , “Philip, Nudging farmers to reduce water pollution from nitrogen fertilizer,” Food Policy, Elsevier, 2023, vol. 120(C).

[6] T. Kang , Y. Shin , M. Shin , D. Lee , K. J. Lim , J. Kim , Water 2024, 16, 1604.

[7] M. J. Scholz , D. R. Obenour , E. S. Morrison , J. J. Elser , Nat. Sustain. 2025, 3, 1.

[8] J. J. Beaulieu , T. DelSontro , J. A. Downing , Nat. Commun. 2019, 10, 1. 30914638 10.1038/s41467-019-09100-5 PMC6435651

[9] A. Siciliano , C. Limonti , G. M. Curcio , R. Molinari , Sustainability 2020, 12, 7538.

[10] C. He , Y. Huang , Q. Shao , F. Kong , D. Zheng , X. Qiu , Int. J. Biol. Macromol. 2025, 296, 139679. 39793840 10.1016/j.ijbiomac.2025.139679

[11] Y. Oladosu , M. Y. Rafii , F. Arolu , S. C. Chukwu , M. A. Salisu , I. K. Fagbohun , T. K. Muftaudeen , S. Swaray , B. S. Haliru , Horticulturae 2022, 8, 605.

[12] A. Morales , J. Labidi , P. Gullón , G. Astray , Curr. Opin. Green Sustain. Chem. 2021, 29, 100436.

[13] H. Yang , F. Zhang , Y. Chen , Y. Xie , R. Wang , Y. He , P. Song , Int. J. Biol. Macromol. 2025, 291, 138969. 39708874 10.1016/j.ijbiomac.2024.138969

[14] Y. Chen , Y. Xu , A. El Idrissi , T. Long , Y. Liu , L. Lu , Int. J. Biol. Macromol. 2025, 310, 143257. 40250668 10.1016/j.ijbiomac.2025.143257

[15] S. Zhang , X. Wang , C. Wu , B. Liu , D. Tian , C. Tian , W. Li , Int. J. Biol. Macromol. 2025, 288, 138498. 39647732 10.1016/j.ijbiomac.2024.138498

[16] K. Nandal , V. Vaid , Rahul , P. Saini , Devanshi , R. K. Sharma , V. Joshi , R. Jindal , H. Mittal , Ind. Crops Prod. 2025, 225, 120587.

[17] S. Panpinit , P. Jumpapaeng , S. Prasertsri , P. Suwanakood , S. Nanan , S. Saengsuwan , J. Indus. Eng. Chem. 2025, 142, 392.

[18] M. Wu , J. Lu , Y. Zhang , Z. Ling , R. Lu , J. Zhu , Y. Li , Y. Cai , H. Xiang , Z. Zhang , B. Yu , Int. J. Biol. Macromol. 2025, 306, 141296. 40010470 10.1016/j.ijbiomac.2025.141296

[19] L. Ma , Y. Song , Z. Li , D. Zhang , K. Li , Q. Duan , H. Xie , X. Yu , L. Yu , Ind. Crops Prod. 2025, 226, 120648.

[20] S. Khanam , S. K. Ray , R. H. Bhuiyan , S. Sultana , N. Sharmin , Q. Ehsan , Ind. Crops Prod. 2025, 224, 120380.

[21] Y. Zhang , Y. Sun , H. Li , M. Niu , X. Wang , Z. Wang , Y. Guo , Colloids Surf. A Physicochem. Eng. Asp 2025, 706, 135800.

[22] Manu , D. Kumar , R. K. Gupta , Ind. Crops Prod. 2024, 219, 119029.

[23] R. Kaur , R. Sharma , G. K. Chahal , Chem. Pap. 2021, 75, 4465.

[24] R. Patel , P. Dhar , A. Babaei‐Ghazvini , M. Nikkhah Dafchahi , B. Acharya , Bioresour. Technol. Rep. 2023, 22, 101463.

[25] E. Dembech , G. Sotgiu , A. Donnadio , S. Buoso , G. Dolci , M. Jo , F. A. Nichilo , V. Sinisi , RSC Adv. 2025, 15, 5344. 39967896 10.1039/d4ra08521c PMC11833289

[26] S. Milad , S. E. Saleh , M. M. Aboulwafa , N. A. Hassouna , Arch. Pharmac. Sci. Ain Shams Univ. 2023, 7, 303.

[27] K. Y. Li , H. Y. Xu , Y. R. Liu , W. Zhong , Y. C. Jin , W. J. Wu , Int. J. Biol. Macromol. 2024, 282, 136786. 39442847 10.1016/j.ijbiomac.2024.136786

[28] F. Bucciol , P. Quagliotto , L. Tedesco , E. Calcio Gaudino , G. Cravotto , S. Tabasso , ACS Sustain. Chem. Eng. 2024, 13, 343.

[29] F. Fu , J. Luo , L. Zhao , F. Yang , N. Wang , PLoS One 2024, 19, 0296366. 10.1371/journal.pone.0296366 PMC10760773 38165910

[30] H. Ji , S. Y. H. Abdalkarim , Y. Shen , X. Chen , Y. Zhang , J. Shen , H. Y. Yu , Int. J. Biol. Macromol. 2024, 278, 134618. 39151851 10.1016/j.ijbiomac.2024.134618

[31] C. He , Y. Huang , Q. Shao , F. Kong , D. Zheng , X. Qiu , Int. J. Biol. Macromol. 2025, 296, 139679. 39793840 10.1016/j.ijbiomac.2025.139679

[32] Y. Liu , L. Cao , L. Wang , Y. Qi , Y. Zhao , H. Lu , L. Lu , D. Zhang , Z. Wang , H. Zhang , Molecules 2024, 29, 1699. 38675519 10.3390/molecules29081699 PMC11051779

[33] Z. Wang , A. Abbas , H. Sun , H. Jin , T. Jia , J. Liu , D. She , Int. J. Biol. Macromol. 2023, 242, 124862. 37210049 10.1016/j.ijbiomac.2023.124862

[34] G. J. Jiao , P. Peng , S. L. Sun , Z. C. Geng , D. She , Int. J. Biol. Macromol. 2019, 127, 544. 30660565 10.1016/j.ijbiomac.2019.01.076

[35] H. Wang , X. Chen , L. Zhang , Z. Li , X. Fan , S. Sun , Ind. Crops Prod. 2021, 166, 113481.

[36] T. Li , Z. Tong , Q. Zheng , H. Bao , A. Partow , S. Meng , L. Li , Y. C. Li , ACS Sustain. Chem. Eng. 2021, 9, 10468.

[37] J. Yu , D. Wang , N. Geetha , K. M. Khawar , S. Jogaiah , M. Mujtaba , Carbohydr. Polym. 2021, 261, 117904. 33766382 10.1016/j.carbpol.2021.117904

[38] F. A. Mesas , M. C. Terrile , M. X. Silveyra , A. Zuñiga , M. S. Rodriguez , C. A. Casalongué , J. R. Mendieta , Plant Pathol. J. 2021, 37, 533. 34897246 10.5423/PPJ.OA.06.2021.0090 PMC8666248

[39] N. R , R. E , H. S , S. V , G. S. R , J. D , J. Nanopart. Res. 2025, 27, 1.

[40] R. S. Riseh , M. G. Vazvani , J. F. Kennedy , Int. J. Biol. Macromol. 2023, 252, 126483. 37625747 10.1016/j.ijbiomac.2023.126483

[41] D. Prajapati , A. Pal , C. Dimkpa , Harish , U. Singh , K. A. Devi , J. L. Choudhary , V. Saharan , Carbohydr. Polym. 2022, 288, 119356. 35450625 10.1016/j.carbpol.2022.119356

[42] T. Jamnongkan , S. Kaewpirom , J. Polym. Environ. 2010, 18, 413.

[43] T. V. de Oliveira , P. A. V. de Freitas , C. C. Pola , J. O. R. da Silva , L. D. A. Diaz , S. O. Ferreira , N. Soares , F. F. de , Food Packag Shelf Life 2020, 24, 100459.

[44] P. Nooeaid , P. Chuysinuan , W. Pitakdantham , D. Aryuwananon , S. Techasakul , D. Dechtrirat , J. Polym. Environ. 2021, 29, 552.

[45] E. Food , S. Authority , EFSA J. 2014, 12, 3820.

[46] J. Park , J. Lee , Y.‐M. Kim , M. J. Kang , H.‐J. Suh , J. Lee , H.‐S. Lee , C Lee , Food Sci. Biotechnol. 2022, 31, 797. 35720465 10.1007/s10068-022-01102-2 PMC9203636

[47] J. Nowicki , A. Dzieniszewska , E. Sabura , G. Rzepa , M. Muszyński , K. Komosińska , Mater. Chem. Phys. 2025, 339, 130605.

[48] G. J. Jiao , P. Peng , S. L. Sun , Z. C. Geng , D. She , Int. J. Biol. Macromol. 2019, 127, 544. 30660565 10.1016/j.ijbiomac.2019.01.076

[49] Y. Matsushita , H. Sano , M. Imai , T. Imai , K. Fukushima , J. Wood Sci. 2007, 53, 67.

[50] C. Li , Y. Li , Q. Li , J. Duan , J. Hou , Q. Hou , S. Ai , H. Li , Y. Yang , Sci. Total Environ. 2021, 788, 147812. 34023609 10.1016/j.scitotenv.2021.147812

[51] Y. Ou , M. Tian , J. Mater. Chem. B 2021, 9, 7955. 34611684 10.1039/d1tb01363g

[52] T. Wang , M. Turhan , S. Gunasekaran , Polym. Int. 2004, 53, 911.

[53] R. V. Gadhave , P. A. Mahanwar , P. T. Gadekar , Des. Monomers Polym. 2019, 22, 164. 31692861 10.1080/15685551.2019.1678222 PMC6818112

[54] L. A. Worku , M. G. Tadesse , A. Bachheti , D. P. Pandey , A. K. Chandel , A. W. Ewuntu , R. K. Bachheti , Int. J. Biol. Macromol. 2024, 254, 127644. 37879578 10.1016/j.ijbiomac.2023.127644

[55] S. Bhardwaj , N. K. Bhardwaj , Y. S. Negi , Cellulose 2020, 27, 5337.

[56] T. S. G. Wudil , L. S. Lawal , F. A. Adedeji , Academia Green Energy 2017, 2, 7659.

[57] E. Campos , P. Coimbra , M. H. Gil , Polym. Bull. 2013, 70, 549.

[58] A. A. Khan , J. K. Nayak , B. U. Amin , M. Muddasar , M. Culebras , V. V. Ranade , M. N. Collins , Int. J. Biol. Macromol. 2024, 281, 136292. 39368579 10.1016/j.ijbiomac.2024.136292

[59] G. A. Avci , E. Avci , G. Özlük , Ş. C. Cevher , Hittite J. Sci. Eng. 2020, 7, 45.

[60] M. B. Jalageri , G. C. M. Kumar , Polym. Bull. 2024, 81, 10915.

[61] Z. Zhang , C. Zhao , X. Tang , A. Yan , Y. Luo , F. Yang , Y. Wang , Eur. Phys. J. Plus 2024, 139, 1.

[62] B. Wang , D. Qiu , Y. Gu , Z. Shan , R. Shi , J. Luo , S. Qi , Y. Wang , B. Jiang , Y. Jin , J. Biores. Bioprod. 2025, 10, 62.

[63] L. Wu , S. Huang , J. Zheng , Z. Qiu , X. Lin , Y. Qin , Int. J. Biol. Macromol. 2019, 140, 538. 31437505 10.1016/j.ijbiomac.2019.08.142

[64] A. Morales , J. Labidi , P. Gullon , J. Indus. Eng. Chem. 2020, 81, 475.

[65] H. Dai , H. Zhang , L. Ma , H. Zhou , Y. Yu , T. Guo , Y. Zhang , H. Huang , Carbohydr. Polym. 2019. 10.1016/j.carbpol.2019.01.014 30732825

[66] H. Ahmed , M. A. R. Noyon , M.d. E. Uddin , M. M. Rafid , M.d. S. Hosen , R. K. Layek , Cleaner Chem. Eng. 2025, 11, 100157.

[67] L. García‐Cruz , C. Casado‐Coterillo , J. Iniesta , V. Montiel , Á. Irabien , J. Appl. Polym. Sci. 2015, 132, 42240.

[68] M. P , P. Murugan K , K. C , S. B. Kameswari K , S. D , S. G , S. G , J. Environ. Manag. 2025, 381, 125204. 10.1016/j.jenvman.2025.125204 40179465

[69] Y. Dou , X. Wang , Z. Liu , F. Kong , S. Wang , J. Appl. Polym. Sci. 2024, 141, 54910.

[70] W. Yang , E. Fortunati , F. Bertoglio , J. S. Owczarek , G. Bruni , M. Kozanecki , J. M. Kenny , L. Torre , L. Visai , D. Puglia , Carbohydr. Polym. 2018, 181, 275. 29253973 10.1016/j.carbpol.2017.10.084

[71] M. V. Ramlogan , A. A. Rouff , J. Therm. Anal. Calorim. 2016, 123, 145.

[72] D. Menezes‐Blackburn , C. Paredes , H. Zhang , C. D. Giles , T. Darch , M. Stutter , T. S. George , C. Shand , D. Lumsdon , P. Cooper , R. Wendler , L. Brown , M. Blackwell , C. Wearing , P. M. Haygarth , Environ. Sci. Technol. 2016, 50, 11521. 27700099 10.1021/acs.est.6b03017

[73] C. Ling , F. Liu , Z. Pei , X. Zhang , M. Wei , Y. Zhang , L. Zheng , J. Zhang , A. Li , B. Xing , Sci. Rep. 2015, 5, 1. 10.1038/srep09944 PMC4649996 25962970

[74] C. K. Choudhury , S. Roy , Soft Matter 2013, 9, 2269.

[75] E. Priya , J. Sharma , S. Sarkar , P. K. Maji , J. Environ. Chem. Eng. 2025, 13, 116716.

[76] Y. Zhang , Y. Sun , H. Li , M. Niu , X. Wang , Z. Wang , Y. Guo , Colloids Surf. A Physicochem. Eng. Asp. 2025, 706, 135800.

[77] S. Zhang , S. Ma , Q. Zhu , React. Funct. Polym. 2025, 212, 106257.

[78] M. Wu , J. Lu , Y. Zhang , Z. Ling , R. Lu , J. Zhu , Y. Li , Y. Cai , H. Xiang , Z. Zhang , B. Yu , Int. J. Biol. Macromol. 2025, 306, 141296. 40010470 10.1016/j.ijbiomac.2025.141296

[79] C. Cao , T. Huo , P. Liu , J. Long , Y. Ma , S. I. Jahan , T. T. Manjoro , F. Dong , Int. J. Biol. Macromol. 2025, 305, 141276. 39984089 10.1016/j.ijbiomac.2025.141276

[80] M. Paswan , S. Patel , V. Prajapati , B. Z. Dholakiya , Int. J. Biol. Macromol. 2023, 253, 126979. 37739290 10.1016/j.ijbiomac.2023.126979

[81] A. A. Khan , J. K. Nayak , B. U. Amin , M. Muddasar , M. Culebras , V. V. Ranade , M. N. Collins , Int. J. Biol. Macromol. 2024, 281, 136292. 39368579 10.1016/j.ijbiomac.2024.136292

[82] K. Lu , R. Abouzeid , Q. Wu , Q. Chen , S. Liu , Giant 2024, 18, 100270.

[83] P. L. Ritger , N. A. Peppas , J. Controlled Release 1987, 5, 37.

[84] S. Arayesh , B. Tanhaei , S. M. Khoshkho , M. N. Shahrak , A. Ayati , S. K. Far , Int. J. Biol. Macromol. 2025, 306, 141295. 39984103 10.1016/j.ijbiomac.2025.141295

[85] X. Tan , G. Dong , Z. Shi , D. Zhang , D. Liu , Y. Wang , J. Li , P. Wang , W. Zhang , Int. J. Biol. Macromol. 2025, 319, 145572. 40578638 10.1016/j.ijbiomac.2025.145572

