# Supplementary material for: Aminated Lignin‐Reinforced Biopolymer Hydrogels for Sustained Phosphate Delivery via Struvite Encapsulation in Acidic Environments
Source: Glob Chall. 2025 Sep 24;9(10):e00288. doi: 10.1002/gch2.202500288 (PMC12519428; doi:10.1002/gch2.202500288)
Supplement: Supplementary file 1 — Supporting Information [file GCH2-9-e00288-s001.docx]

**SUPPLEMENTARY INFORMATION**

**Aminated Lignin–Reinforced Biopolymer Hydrogels for Sustained Phosphate Delivery via Struvite Encapsulation in Acidic Environments**

Abrar Ali Khan^a^, Arvind Singh Chandel^a^, Vivek V. Ranade^b^, Maurice N. Collins^a,c,d^

^a^ Stokes Laboratories, School of Engineering, Bernal Institute, University of Limerick, Limerick V94 T9PX, Ireland

^b^ Multiphase Reactors and Intensification Group, Bernal Institute, University of Limerick, Limerick V94 T9PX, Ireland

^c^ Health Research Institute and AMBER University of Limerick, Limerick V94 T9PX, Ireland

^d^ SFI Centre for Advanced Materials and BioEngineering Research, Dublin D02 PN40, Ireland

Table S **1**| Summary table of Precursors for Hydrogel Matrix

| Sample Code | PVA (g) | Chitosan (g) | Aminated Lignin (g) | Cross Linker Glutaraldehyde (1.65%, v/v) | Total Volume (mL) | Struvite (mg) |
| --- | --- | --- | --- | --- | --- | --- |
| P-Chi | 0.6 | 0.2 | 0 | 3 mL | 20 | -- |
| P-Chi-AL_1_ | 0.6 | 0.2 | 0.1 | 3 mL | 20 | -- |
| P-Chi-AL_2_ | 0.6 | 0.2 | 0.2 | 3 mL | 20 | -- |
| P-Chi-AL_3_ | 0.6 | 0.2 | 0.3 | 3 mL | 20 | -- |
| St-P-Chi | 0.6 | 0.2 | 0 | 3 mL | 20 | 100 |
| St-P-Chi-AL_1_ | 0.6 | 0.2 | 0.1 | 3 mL | 20 | 100 |
| St-P-Chi-AL_2_ | 0.6 | 0.2 | 0.2 | 3 mL | 20 | 100 |
| St-P-Chi-AL_3_ | 0.6 | 0.2 | 0.3 | 3 mL | 20 | 100 |


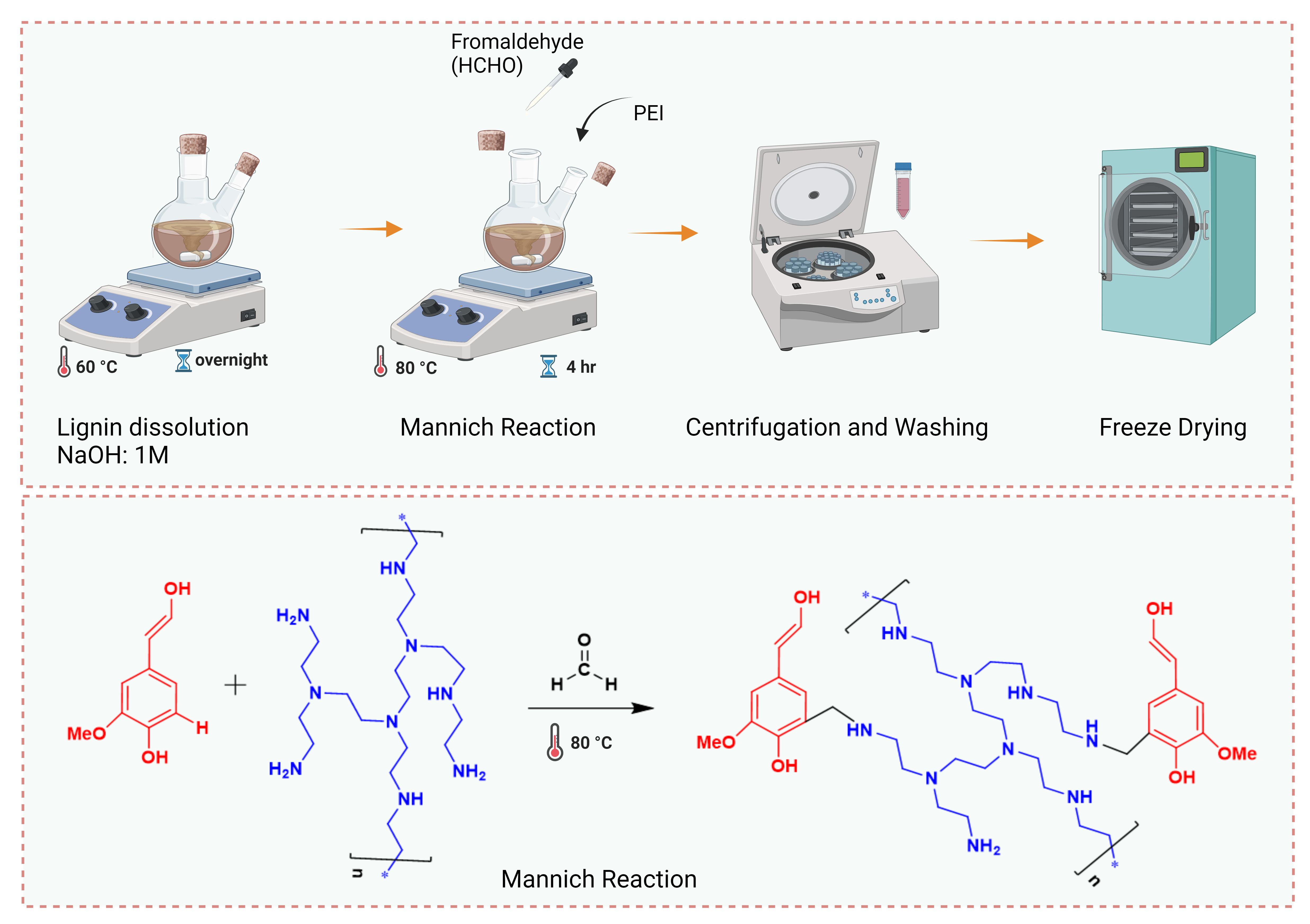


Figure S 1| Schematic representation of lignin amination procedure and the corresponding reaction mechanism


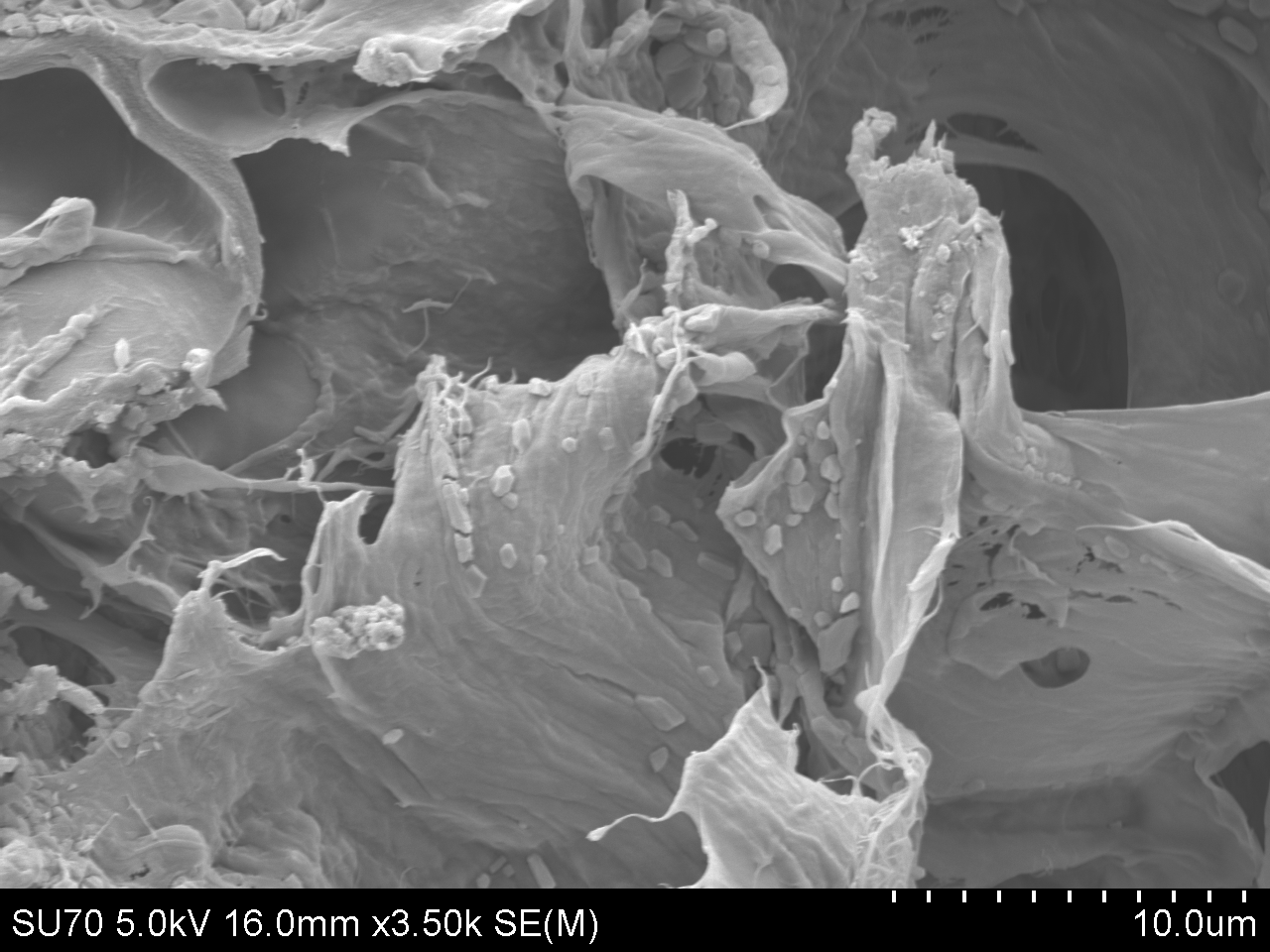

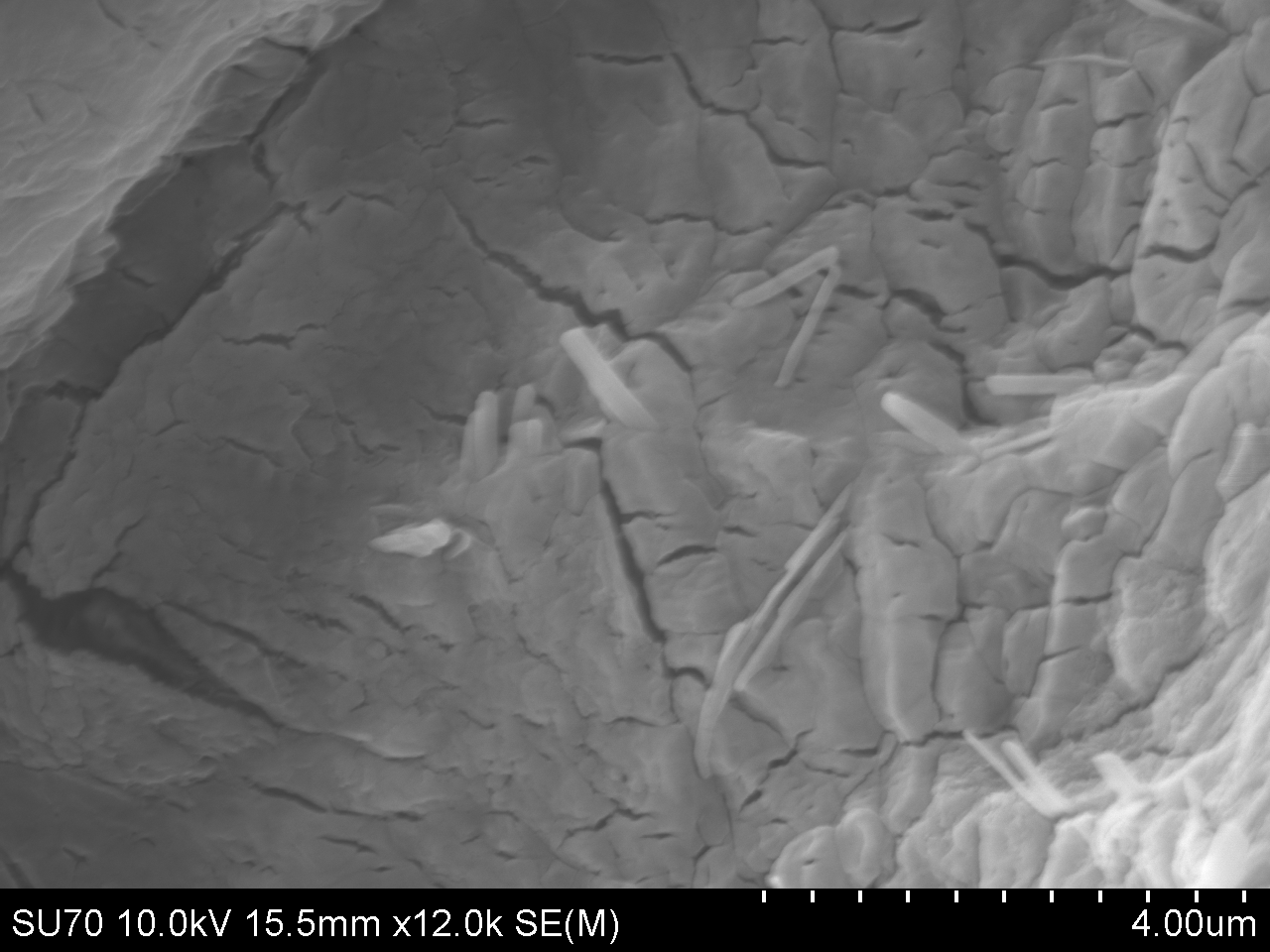


Figure S 2| SEM images of struvite loaded hydrogels at higher magnifications


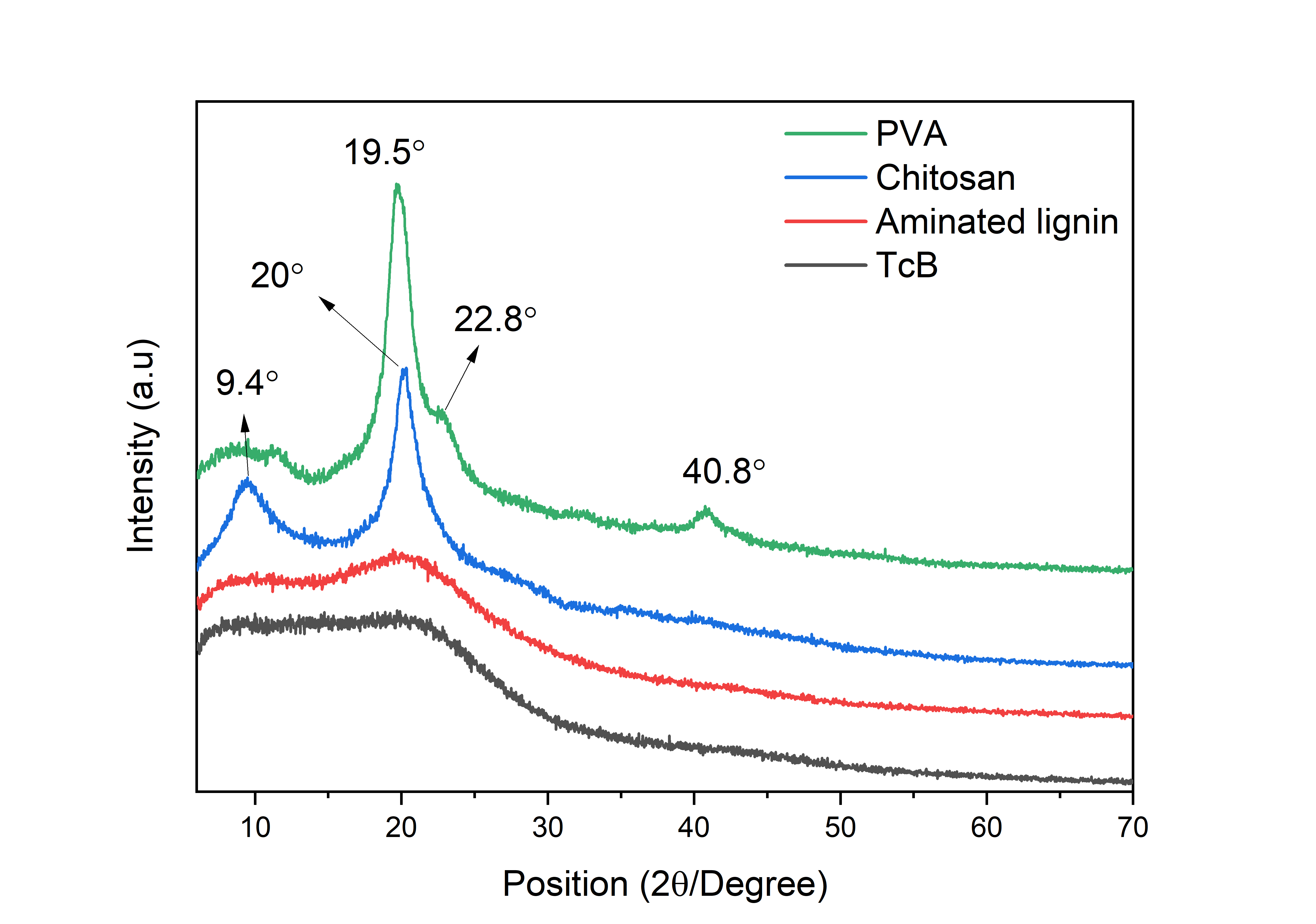


Figure S 3| XRD of hydrogel precursors including aminated lignin


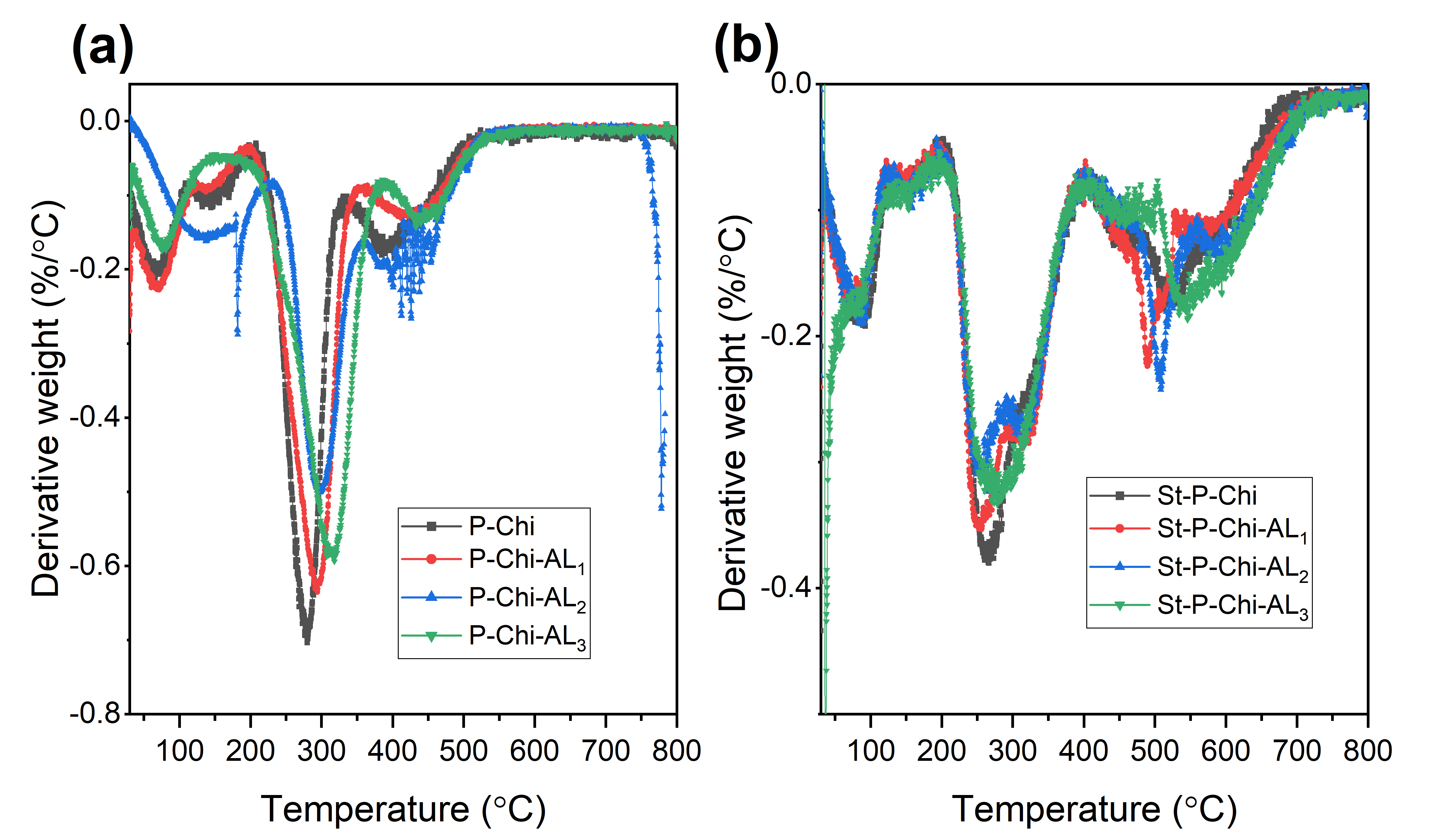


Figure S 4| DTG curves of pristine hydrogel (a), and struvite ladened hydrogel (b).


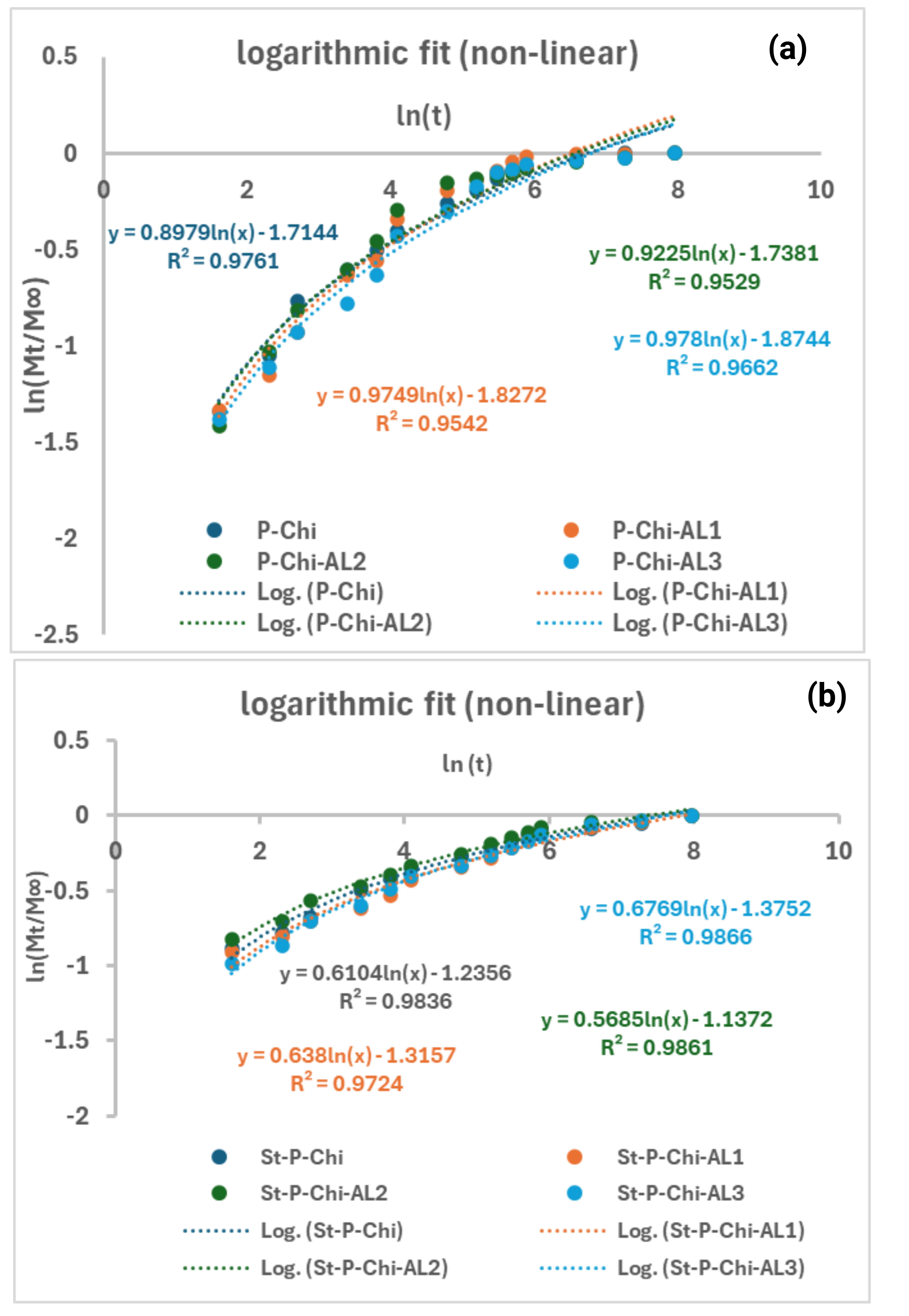


Figure S 5| Fitting the experimental swelling data with Korsmeyer-Peppas nonlinear kinetic model, pristine hydrogel (a), and struvite loaded hydrogel (b)





Figure S 6| Fitting the experimental phosphate release data (both linear and non-linear) with Korsmeyer-Peppas kinetic model (a), Higuchi Model (b), Zero-order (c), and First-order Model (d)
